# Cyclodextrin-Based Polymeric Drug Delivery Systems for Cancer Therapy

**DOI:** 10.3390/polym15061400

**Published:** 2023-03-11

**Authors:** Xuebing Li, Junda Liu, Neng Qiu

**Affiliations:** 1 Institute of Fundamental and Frontier Sciences, University of Electronic Science and Technology of China, Chengdu 611731, China; 2 Department of Chemical Engineering, College of Materials and Chemistry and Chemical Engineering, Chengdu University of Technology, Chengdu 610059, China

**Keywords:** cyclodextrin, polyrotaxane, conjugate, polymer, crosslink, copolymer

## Abstract

Cyclodextrins (CDs) are one of the most extensively studied cyclic-oligosaccharides due to their low toxicity, good biodegradability and biocompatibility, facile chemical modification, and unique inclusion capacity. However, problems such as poor pharmacokinetics, plasma membrane disruption, hemolytic effects and a lack of target specificity still exist for their applications as drug carriers. Recently, polymers have been introduced into CDs to combine the advantages of both biomaterials for the superior delivery of anticancer agents in cancer treatment. In this review, we summarize four types of CD-based polymeric carriers for the delivery of chemotherapeutics or gene agents for cancer therapy. These CD-based polymers were classified based on their structural properties. Most of the CD-based polymers were amphiphilic with the introduction of hydrophobic/hydrophilic segments and were able to form nanoassemblies. Anticancer drugs could be included in the cavity of CDs, encapsulated in the nanoparticles or conjugated on the CD-based polymers. In addition, the unique structures of CDs enable the functionalization of targeting agents and stimuli-responsive materials to realize the targeting and precise release of anticancer agents. In summary, CD-based polymers are attractive carriers for anticancer agents.

## 1. Introduction

As the second leading cause of death across the world, cancer was estimated to result in 9.6 million deaths in 2018, according to the World Health Organization (WHO) [1]. In the past several decades, remarkable progress has been achieved in cancer therapy, and the survival rate of several types of cancers has been improved, accounting for early detection and new treatment. However, a complete cure for cancer is still far away from reality. In recent years, various research efforts have been made in chemotherapy, immunotherapy, cell therapy and gene therapy to improve their therapeutic efficacy for cancer treatment. Chemotherapy is a principal therapeutic for cancer treatment. However, most clinically approved small-molecule anticancer drugs are poorly water-soluble, with low tumor selectivity and high systemic toxicity, limiting their therapeutic efficacy [2]. The emergence of delivery systems based on polymers, lipids, inorganic carriers and hydrogels has had a positive impact on improving the treatment efficacy of chemotherapeutics. However, the development of delivery systems for cancer therapy still suffers from a lack of therapeutic efficacy, inadequate safety and limited delivery components/materials approved by the agencies.

**Figure 1:**
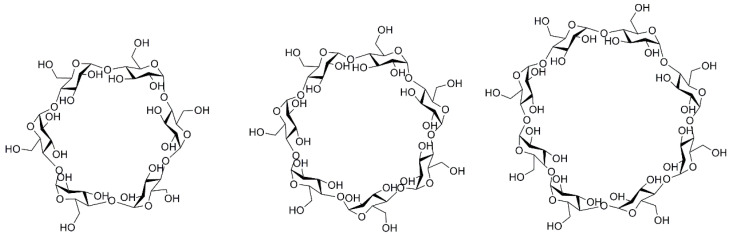
Chemical structure of α-CD, β-CD and γ-CD.

Cyclodextrins (CDs) have attracted great attention for the delivery of anticancer drugs due to their nontoxicity, low immunogenicity, excellent aqueous solubility, facile modification and acceptable price with wide availability. In addition, CDs have been approved as pharmaceutical excipients in many countries and have been safely used clinically for many decades. CDs are a class of macrocyclic oligosaccharides composed of six or more glucose subunits linked by α-1,4 glycosidic bonds. Natural CDs can be classified into three categories of α-, β- and γ-CD containing 6, 7 and 8 glucose units, respectively (Figure 1). They are toroidal in shape with a hydrophobic inner cavity and hydrophilic surface. Lipophilic anticancer drugs can be encapsulated in the hydrophobic inner cavity of CD molecule by host–guest complexation effects. Therefore, the water solubility of the guest molecule could be enhanced due to the hydrophilic surface of the CD molecule. Many studies have shown that complexation with CD could significantly enhance the solubility and stability of anticancer drugs [3,4,5]. Although the inclusion complex based on CDs provides promising platforms for chemotherapeutics, only improving the solubility and stability of anticancer drugs cannot meet the requirements in cancer therapy. To enhance therapeutic efficacy with minimized side effects, an ideal anticancer delivery system should transport drugs to tumor sites while avoiding the delivery of drugs to normal tissues, prevent degradation in the blood stream with controlled release behavior and be able to across biological barriers. The combination of polymer chemistry and CD chemistry has gained great attention, since these CD-based polymeric delivery systems could provide better therapeutic effects in cancer therapy [6]. The aim of this review is to summarize and highlight some of the CD-based polymeric drug delivery system potential for cancer treatment.

## 2. CD-Based Polyrotaxanes

**Figure 2:**
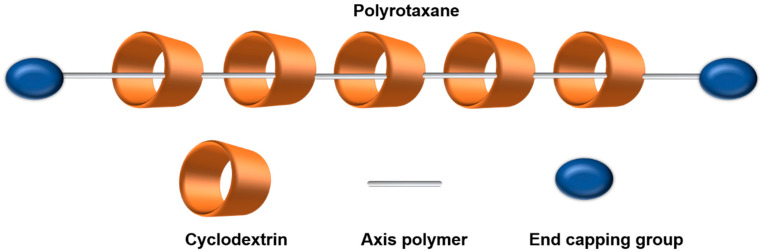
Schematic illustration of polyrotaxane.

The name of rotaxane comes from the Latin words for wheel (rota) and axle (axis). A rotaxane is a topological supramolecule consisting of a ring-like molecule that is threaded onto an inner linear molecule chain. A polyrotaxane (PR) is a class of molecular component in which multiple cyclic molecules are threaded around a linear macromolecule with bulky groups at the ends (Figure 2) [7]. The end groups can prevent the PR from dethreading. Recently, CD-based PRs have attracted great attention owing to their unique biocompatibility and accessibility. For their cyclic structure and excellent complexation properties, inclusion complexes formed by the host–guest interaction between CD molecules and polymer axes readily obtain these necklace-like interlocked supermolecules [8,9].

The PRs with CDs as hosts and ethylene glycol (PEG)-based polymers as guests are the most extensively studied CD PRs. CDs and PEG are classified as generally regarded as safe by the US FDA, and both have been approved by regulatory agencies in many countries. In addition, biocompatible bulky blocking caps (e.g., tyrosine) could be introduced at the ends with a cleavable bond, and thus PRs could be dethreaded and eventually cleared from the body [10,11,12]. Therefore, CD-based PRs are attractive drug carriers for chemotherapeutics. Furthermore, plenty of hydroxy groups on the CD molecules of PRs can be easily modified and used as multivalent drug carriers.

### 2.1. Anticancer Drug–Polyrotaxane Conjugate

Drugs directly conjugated to carriers with cleavable linkage that can be enzymatically degraded in the body have attracted great attention for cancer treatment. The abundant hydroxy groups on the surface of CDs allow therapeutic agents with functional group to be easily linked to the backbone of PR by hydrolysable bonds. The solubility of conjugated drugs could be significantly enhanced after linking to PRs due to their hydrophilic nature. In addition, the attached drugs with covalent bonds could exhibit a long sustaining release behavior. Furthermore, many anticancer drugs are water-insoluble, endowing these drug-PR conjugates with amphiphilicity and self-assembling into core–shell nanoparticles in which lipophilic drugs are located in the core and PRs exist on the shell. Moreover, due to the large number of hydroxy groups on CD-based PRs, high drug loading capacity could be easily obtained in the conjugates.

Camptothecin (CPT) is a highly effective anticancer drug. However, the clinical application has been considerably hindered due to its low aqueous solubility, instability at physiological conditions, and severe side effects such as gastrointestinal toxicities. Moon and colleagues synthesized PR-CPT conjugates via hydrolysable ester linkage [13]. PEG and α-CD were chosen to prepare the PR with L-Tyr (tyrosine) as the end groups; CPT was covalently attached to the CD surface through ester bond with a succinic anhydride as a spacer. To further increase the therapeutic efficacy and reduce the toxicity, LMWP (a cell-penetrating peptide) was attached to the PR-CPT conjugates to allow for tumor site targeting and enhanced the intracellular uptake of CPT. In addition to achieving a desirable drug loading content of ~8%, LMWP–PR–CPT conjugates showed a seven-fold increase in water solubility and a prolonged release profile up to >7 days. Drug molecules can also be conjugated to PRs through stimulus-sensitive linkages. These stimuli-modified drug–PR conjugates are able to release anticancer drugs at specific disease sites, triggered by internal tumor microenvironment stimuli (e.g., pH, redox environment and hypoxia) and external stimuli (e.g., light, thermo and magnetic fields) [14]. Bai et al. developed a reduction-responsive CPT-PR conjugate through the introduction of a disulfide bond to realize the stimuli release of the active drug molecule. In this conjugate, α-CDs were threaded onto PEG4000 with 9-anthracenemethanol as the terminal stopper. CPT with disulfide linker was grafted onto CDs via atom transfer radical polymerization (ATRP), and a hydrophilic poly(ethylene glycol)methyl ether methacrylate (OEGMA) chain was also linked onto the PR backbone. The resulting amphipathic PC-PCPT-bPOEGMA (PCCO) could self-assemble into monodispersed micelles with a diameter of 113.9 nm. The PCCO with a disulfide bond showed an effective reduction release (>85%) in the presence of DTT. The PCCO micelles exhibited a time-dependent cellular uptake behavior and showed favorable blood compatibility on a mouse model [15].

Paclitaxel (PTX) is an extensively studied chemotherapeutic agent that has been widely used clinically as a first or second treatment option for many cancers since its approval by the US FDA in 1992. PTX stabilizes microtubules by binding to the β-tubulin subunit and preventing its depolymerization, thus blocking cell cycle progression and suppressing the growth of cancer cells [16,17]. Yu and coworkers synthesized PR-PTX conjugate through a diaminotri (ethylene glycol) spacer via an ester linkage. Unlike α-CD PRs with PEG as the polymer axle, β-CD rings were threaded onto poly(propylene glycol) (PPG) with β-CD as the end-capping group in this study. The resulting PR-PTX conjugate exhibited a high drug loading content of approximately 29% and showed a sustained release profile. In vivo results also attained a prolonged release behavior, with t_1/2_ calculated to be about 5.8 h compared to 18.8 min reported for Taxol. The in vivo antitumor effect was tested in a H22 tumor-bearing mice model; PR-PTX (78.7%) exhibited a significantly higher tumor growth inhibition rate in comparison with Taxol (30.7%) at a dose of 10 mg/kg. In addition, the lifetime of tumor-bearing mice was prolonged by PR-PTX treatment when compared to Taxol treatment with a median survival time of >61 days and 49 days, respectively. Furthermore, after conjugating with PR, PTX was more likely to accumulate in tumor tissues and showed low toxicity to normal tissues [18]. This group also developed a PR-PTX conjugate based on α-CD and PEG. PEG with primary -NH_2_ groups was utilized as the axle with Z-PHE-OH (N-carbobenzoxy-L-phenylalanine) as the blocking end groups. A polymer chain with PHEMA (Poly(2-hydroxyethyl methacrylate)) and PCB-tBu (poly(2-tert-butoxy-N-(2-(methacryloyloxy)ethyl)-N,N-dimethyl-2-oxoethanaminium)) as each block arm was introduced to the PRs via ATPR to obtain PR-PHEMA-PCB. PTX was covalently grafted to the PHEMA chain through ester linkage. The resulting PTX-loaded PR-PHEMA-PCB (PR-PTX-PCB) was spherical in shape, with a particle size of 3.5 nm. PR-PTX-PCB enhanced the water solubility of PTX with a drug loading content of 6.6%. PR-PTX-PCB exhibited a significantly prolonged release property (half-life: 7.7 h) in blood circulation when compared to commercially available Taxol (half-life: 18.8 ± 1.5 min). In addition, PR-PTX-PCB treatment showed increased antitumor effectiveness, with TGI (tumor growth inhibition) determined to be 67.0% versus 49.2% of the Taxol treatment. The animal survival time of the PR-PTX-PCB-treated group was also prolonged compared with the Taxol-treated group and the PR-PHEMA-PCB-treated group [19].

### 2.2. Polyrotaxane-Based Nanoparticles as Anticancer Drug Carriers

Nanoparticles have been extensively studied as delivery vehicles for chemotherapeutics due to their unique physiochemical propertie. In the past few decades, great efforts have been made to use biocompatible nanoparticles for cancer treatment. Nanoparticles can prolong the drug half-life in blood circulation, passively accumulate in neoplastic sites and decrease toxic effects for normal tissues [20]. CD-based PRs have been reported to form nanoparticles through the crystallization of PRs. However, these PR-nanoassembled particles are not able to trap lipophilic anticancer drugs due to their compact architectures [21]. To acquire PR-based nanoparticles suitable for encapsulating anticancer drugs, chemical modification plays a key role.

Generally, water solubility is particularly important for the application of pharmaceutical materials. However, because of the strong intramolecular hydrogen bond formed between hydroxy groups of threaded CDs, the solubility of PRs is poor in aqueous solutions. To improve the solubility of PRs, chemical modification could efficiently modulate its water solubility. In general, there are two approaches for chemical modification: one approach is to modify the hydroxy group of threaded CDs; another approach is to introduce terminal functional groups at the ends of PEG chains. The assembly of nanoparticles could be regulated by the modulation of the ratio of hydrophilic and hydrophobic components in amphiphilic materials [22,23]. Therefore, self-assembled nanoparticles could be acquired if proper functional groups are introduced to PR.

Dam and Caruso developed a PR consisting of three alkyne groups conjugated at the ends of PEG via disulfide bonds (PR-SS-3A). PEG-azide could be successfully connected to alkyne groups through Cu(I)-catalyzed alkyne-azide click reaction. The triblock polymer of PR could self-assemble into core–shell particles with a size of 100 nm. The PR nanoparticles were capable of encapsulating small molecules, such as pyrene and calcein [24]. Interestingly, if α-CDs threaded onto PR-SS-3A were modified with succinic anhydride, stable spherical nanoparticles with a size of 169 nm could be formed [25]. Liu and coworkers developed PR-based nanoparticles to encapsulate anticancer drugs. PEG_2000_ was selected as the polymer axle and cinnamic acid was linked to the terminal groups of the polymer chain as the end-capping agent. The existence of the cinnamic group reduced the number of α-CD molecules threaded on the PEG chain and provided a hydrophobic microenvironment capable of entrapping anticancer drugs. The cinnamic-modified PR (cin-PR) could self-assemble into nanoparticles with a mean diameter of 57 nm. Doxorubicin (Dox) was trapped in the nanoparticles with a high drug loading content of 18.4%. The drug-loaded cin-PR nanoparticles exhibited enhanced anticancer tumor effects in a mice model inoculated with breast cancer cells. The inhibition rate of Dox-loaded nanoparticle treatment was 53% versus 38% for doxorubicin hydrocholoride treatment at a dose of 5 mg/kg. In addition, the cardiac toxicity of Dox was significantly reduced after encapsulating into cin-PR nanoparticles [26]. Methotrexate (MTX) is an antimetabolite that has been utilized for the treatment of various types of cancers [27]. MTX was also entrapped in the cin-PR nanoparticles with a drug loading content as high as 20.8%. The mean particle size of the MTX-loaded cin-PR was about 200 nm with a spindle-like morphology. Cytotoxicity studies showed that MTX-loaded cin-PR nanoparticles exhibited enhanced anticancer activity against HepG2 cell lines [28].

Modification of the hydroxy groups of CDs threaded along the PEG axle is also capable of forming colloidal nanoparticles. Tonegawa and colleagues developed a variety of PRs that consisted of acetylated α-CD threaded onto PEG with different molecular weights. Adamantyl groups were grafted to the ends of PEG. The molecular weight of the PEG axle and the degree of acetylation of α-CD strongly affected the solubility of acetylated PRs (Ac-PRs). Ac-PRs with low degree of acetylation (<35%) can be dissolved in water regardless of the length of the PEG axle, whereas a high degree (>40%) of acetylation of α-CD threaded on a high-molecular-weight PEG (more than 100,000) was self-assembled into colloidal nanoparticles. Interestingly, Ac-PRs with a high degree of acetylation and a PEG axle with a molecular weight of less than 35,000 were prone to precipitate in aqueous solution. The formation of hydrogen bonds or hydrophobic interactions between acetyl groups on α-CD may account for the self-assembly of Ac-PR nanoparticles with a high degree of acetylation and a high molecular weight of PEG (>100,000) axles. Additionally, the Ac-PRs was able to encapsulate hydrophobic PTX and showed potential as drug carriers for anticancer drugs [29]. The hydrophobicity of modified groups on α-CD greatly affected the solubility and self-assembly properties of PRs. Propionylated PRs (Pr-PRs) and butyrylated PRs (Bu-PRs) with degrees of substitution more than 18% and 11% can also form nanoparticles. The morphology of Pr-PR and Bu-PR nanoparticles were spherical, with a mean particle size of 76.5~79.4 nm. Interestingly, the increase in hydrophobicity decreased the CMC value. Ac-PRs were determined to be 258~292 μg/mL, while the CMC values of Pr-PRs and Bu-PRs remarkably decreased to 50.9 μg/mL and 5.12 μg/mL. Additionally, the more hydrophobic acyl groups’ modification obtained higher drug loading content. Pr-PRs showed higher PTX loading content and encapsulation efficiency in comparison with Ac-PRs. However, PRs with more hydrophobic acyl groups such as valeryl or benzyl group modification precipitated in aqueous solution. These studies demonstrate that moderate hydrophobic acyl groups’ modification plays a pivotal role in the formation of nanoparticles and the encapsulation of lipophilic anticancer drugs [30].

### 2.3. Polyrotaxane-Based Delivery System for Cancer Gene Therapy

With the objective to deliver nucleic acids such as RNA [31,32], DNA [33,34] or antisense oligonucleotides [35,36] to cure human diseases, gene therapy has attracted great attention in the past several decades. Since the first gene medicine (Vitravene) approved by FDA in 1998, 40 gene therapy products have been officially approved by drug regulatory agencies worldwide. Several gene medicines such as gendicine, oncorine, rexin-G and Imlygic have been authorized for cancer treatment [34,37]. However, degradation owing to the nucleases in the plasma and an inability to cross the hydrophobic cell membrane due to its hydrophilicity and polyanionic properties limited the clinical application of gene products. In this regard, it is imperative to develop efficient and safe carriers to transfer gene products into the diseased tissues [38,39]. Viral and nonviral vectors are two main carriers utilized in gene therapy [38,40]. Gene therapy based on viral vectors provides high transfection efficiency and stable gene expression [41]. However, several limitations, including immunogenicity, low loading capacity for gene packages and transgene misinsertion risks lead to the development of nonviral vectors. Nonviral vectors have the benefits of better biological safety, low immunogenicity and low mutagenesis compared with viral vectors [42,43].

Cationization of biocompatible components such as liposomes and polymers is an effective strategy to deliver DNA/RNA. Due to its large number of hydroxy groups on CD molecules threaded on PEG axle, PR is easy to be modified with cationic molecules or polycations via biodegradable linkage. In addition, both ends of PEG or the bulky stoppers of PR could also be grafted with polycations to facilitate gene delivery. Yang and colleagues developed several cationic PRs by conjugating linear or branched oligoethylenimine (OEI) to α-CD molecules. Poly[(ethylene oxide)-ran-(propylene oxide)] (P(EO-r-PO)) random copolymer was chosen as the linear axle with 2,4,6-Trinitrobenzene sulfonic acid (TNBS) grafted to the ends to form bulky stoppers. These cationic PRs could self-assemble into nanoparticles with sizes of 100–200 nm and bind pDNA efficiently. Cationic PRs with linear OEI showed lower cytotoxicity when compared to branched PEI with molecular weight of 25 kD in HEK293 and COS7 cells. Additionally, these OEI modified cationic PRs exhibited high gene transfection efficiencies in various of cell lines including HEK293, COS7, BHK-21, SKOV-3, and MES-SA cells. Interestingly, cationic PRs with pentaethylenehexamine modification showed about 10-fold enhancement of transfection efficiency when compared with PEI (25 kD) at N/P ratios of 20–30 [44]. Badwaik et al. reported three cationic PRs (PR^+^) consisted of HP-β-CD(2-hydroxypropyl-β-cyclodextrin) modified with DMEDA (N,N-dimethylaminoethylamine) threaded on Pluronic^®^ copolymers and end capped with cholesterol. These cationic PRs were evaluated for their siRNA delivery efficiency. The siRNA delivered PR^+^ (PR^+^: siRNA complexes) showed comparable or better cell viability and cellular uptake efficiencies (≥90%) than the commercially available transfection L2K. The PR^+^: siRNA complexes formed typical multilamellar spherical nano-aggregate particles with mean diameters of 200 ± 10 nm. Efficient gene silencing (>80%) was obtained in multiple cell lines such as HeLa and NIH 3T3. In addition, effective charge ratio, PO (polypropylene oxide) block length, and the central PO block coverage of the PR^+^ core greatly affect the silencing efficiency. It seems that lower molecular weight and rigid rod-like PR^+^ polymer cores would be beneficial to increased gene-silencing efficiency [45]. Song et al. developed an ABA triblock cationic PR with three ethanolamine-modified poly(glycidyl methacrylate) (PGEAs) grafted onto CDs as a B block and terminal poly(aspartic acid) (PAsp) of the PEG backbone as an A block. The ABA cationic PR (PP-PGEA9) exhibited significantly higher pDNA transfection efficiency in comparison with PEI at their optimal N/P ratios. When the tumor suppressor gene p53 was loaded, PP-PGEA9/p53 could efficiently inhibit the C6 cell migration and proliferation and notably induce cell apoptosis. In the C6 glioma-bearing mice model, PP-PGEA9/p53 significantly suppressed tumor growth. Immunohistochemical analysis demonstrated that the PP-PGEA9/p53-treated group exhibited remarkably more p53 expression than the PBS-treated group [46].

Multiarmed cationic PRs are capable of condensing genes at lower N/P ratios, allowing for less cationic PRs utilization, thus reducing systemic toxicity owing to high doses and off-target effects. Kulkarni and coworkers synthesized a family of multiarmed cationic PRs constructed with branched PEG amine as the axle. TNBS was grafted to the ends of PEG amine as the stopper groups. DMEDA was coupled onto the threaded α-CD through CDI (1,1’-carbonyldiimidazole) activation to produce the cationic PRs (bPRTx^−^). The multiarmed bPRTx^−^ was shown to readily complex siRNA at lower N/P ratios when compared with bPEI. The formed bPRTx^−^/siRNA complexes were spherical with diameter of 100–25 nm in size. In vitro studies showed that the bPRTx^−^ displayed comparable toxicity with bPEI. Additionally, the multiarmed cationic PRs exhibited excellent gene-silencing efficiency, which was similar to the commercially available Lipofectamine 2000 (L2k) and bPEI [47].

The introduction of tumor-targeting agents or stimuli-responsive groups could effectively improve the gene transfection efficiency. For its low toxicity, low immunogenicity, and high affinity to folate receptors (FR) overexpressed in a variety of cancer cells, FA (folic acid) has been widely used as an excellent targeting agent to improve the delivery of anticancer drugs or gene products [48,49]. FA-modified PEG could increase the cell uptake of PR nanoparticles in KB cells [50]. Additionally, FA introduced to cationic PRs could achieve enhanced efficiency of gene delivery. In a PR-based plasmid DNA vector, FA was capped on both ends of the PEG_4000_ axle and threaded within PEI-modified α-CDs; this FA-modified cationic PR was termed as FPP. FPP was able to condense pDNA into small nanoparticles with a diameter of 75–200 nm and zeta potential in the range of 20–30 mV. The FPP/pDNA complex exhibited an excellent safety profile both in vitro and in vivo, which may be partly attributed to the relatively low zeta potential and the presence of PEG in the cationic PR. FPP/pDNA can significantly enhance the transfection efficiency in FR-positive KB cells. A p53 suppressor gene pcDNS3.1-p53 was delivered by FPP. In the KB cell-bearing mice model, the inhibitory effect of the FPP/DNA(p53)-treated group was superior to that of the BPP (Boc-L-tyrosine-terminated PR PEI)/DNA(p53)-, PEI-25 kDa/DNA (p53)- and naked DNA (p53)-treated groups. The apoptotic-associated genes Bax and caspase 9 expressions were greatly upregulated in the FPP/DNA(p53)- and BPP/DNA(p53)-treated group [51]. Stimuli-responsive structures are usually designed to increase the site targeting or intracellular release properties. Wen and colleagues developed a reducible PR-based cationic copolymer (SS-PR) through the introduction of disulfide bonds at the ends of PEG axle. A series of pseudocomb polycations (SS-PR-pDM) with cationic groups were grafted onto α-CD through ATRP of DMAEMA by utilizing SS-PR-Br as a macroinitiator. The disulfide bond-linked SS-PR and SS-PR-pDM facilitated the intracellular release of pDNA. Interestingly, SS-RP-pDM showed enhanced intracellular luciferase gene transfection efficiency when compared to SS-PR in Hela cells. The high cationic density of SS-RP-pDM due to the grafted pDM chains may be beneficial to the penetration of pDNA into nuclei [52].

## 3. CD–Drug Polymeric Conjugate

### 3.1. NLG 207

**Figure 3:**
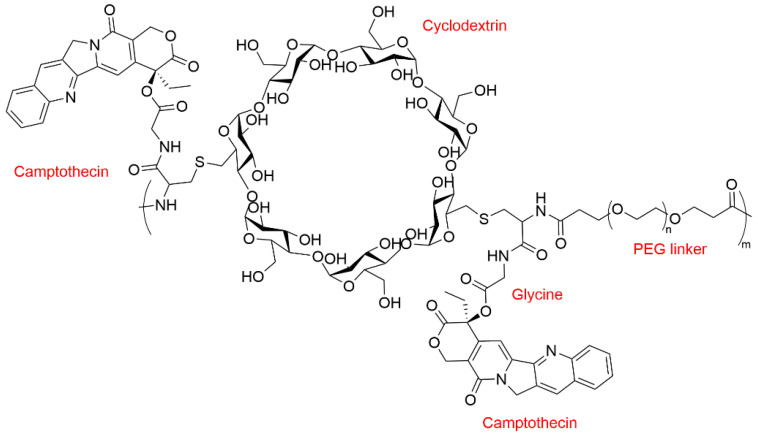
Chemical structure of NLG 207 (CRLX 101).

Due to its abundant hydroxy groups on CDs, it is possible to covalently conjugate anticancer drugs onto CD molecules directly or indirectly. The CD–drug conjugates are comprised of hydrophilic CD components and hydrophobic chemotherapeutic agents, and thus readily form micelles. NLG207 (formerly CRLX101) is a CPT analog connected to CD and PEG blocks with a loading content of 10–12%. In the chemical structure of NLG207, CD and PEG are connected via a biodegradable amino acid linker to form the copolymer backbone; CPT is linked to glycine through an ester linkage at the 20-OH position (Figure 3). NLG207 could self-assemble into nanoparticles with diameters in the range of 20–60 nm and a slightly negative zeta potential. As a result, a more than 1000-fold aqueous solubility increase could be achieved in comparison with the parent drug CPT. The conjugation at the 20-OH position of CPT stabilizes the lactone ring, which is highly susceptible to spontaneous hydrolysis to the inactive carboxylate form at physiologic pH. In addition, the presence of PEG imparts the stealth profile for NLG207, thus preventing RES (reticuloendothelial system) uptake and prolonging the circulation time. Preclinical studies showed that NLG 207 was well tolerated with no observable toxicity, even at a high dose of 240 mg/kg in mice. Drug concentrations in tumor tissues were calculated to be >10^2^ higher than those of SN-38 and >10^4^ higher than those of irinotecan [53,54].

Specifically, glycine linker presented in the conjugate retards the release of the active lactone form of CPT at relatively low pH conditions that would be encountered in an acidic tumor microenvironment (~6.5) and lysosomes after entering cells [55,56,57]. In fact, the release of CPT from NLG 207 was found to be pH-dependent, with longer t_1/2_ values at acidic conditions [58]. Interestingly, esterase activity was not the main reason for the release of CPT from the ester linkage in NLG 207. Phase Ia clinical trials showed that the t_1/2_ of conjugated CPT was about 30 h and that of unconjugated CPT was more than 40 h, respectively [59]. NLG 207 was shown to be able to accumulate in tumor tissues of mouse xenografts and remained in the form of nanoparticles. The release of CPT in mice bearing NCI-H1299 was sustainable for more than 2 weeks, and toposisomerase-1 was significantly inhibited for at least 1 week after one dose of 6 mg/kg [59,60]. Similar results reported by Weiss et al. showed that both NLG 207 nanoparticles and CPT were detectable in a tumor biopsy from a patient with triple-negative breast cancer at day 14 after a single dose of NLG207 (15 mg/m^2^) treatment [61]. In patients with gastric tumors, intact polymer–drug nanoparticles and CPT were also observed in tumor tissues after 24–48 h dosing of 15 mg/m^2^ of NLG207. Meanwhile, no evidence of either nanoparticles or released CPT was shown in adjacent nonneoplastic tissues. The accumulation of NLG 207 in tumor tissues but not in adjacent nonneoplastic tissues demonstrated the high tumor targetability of CPT–polymer conjugate and may provide evidence of the existence of EPR effects in human tumors [62].

Based on its strong anticancer activity in multiple preclinical in vivo studies, several clinical studies have been conducted to investigate the efficacy of NLG207 in various tumors as a single agent or combined with other agents. The first-in-human phase 1/2a clinical trials reported by Weiss et al. in solid malignancies demonstrated that NLG 207 mitigated CPT-associated toxicities such as cystitis. The MTD (maximum tolerated dose) was determined to be 15 mg/m^2^. In patients treated at MTD biweekly, 28 patients of 44 (64%) achieved SD (stable disease), and the median progression-free survival (PFS) was 3.7 months. More encouraging results were observed in NSCLC (nonsmall-cell lung cancer) with PFS of 4.4 months, and 16 patients out of 22 (73%) reported SD [61].

Until recently, multiple clinical trials utilizing NLG207 for the treatment of cancers such as advanced ovarian cancer, gastroesophageal cancer, advanced nonsmall-cell lung cancer and metastatic renal cell carcinoma had been reported in the literature [63,64,65]. Bevacizumab, a humanized IgG1 monoclonal antibody with high-affinity binding to VEGF-A (vascular endothelial growth factor) isoforms, has been approved to be used as a single agent or with other drugs to treat various cancers [66]. Elevated intratumor hypoxia and increased HIF-1α (hypoxia-inducible factor-1α) is attributed to the reduced efficacy of VEGF inhibitors [67]. NLG 207 was reported to be a potent HIF-1α and topoisomerase (topo-1) [68]. Therefore, the combination of NLG 207 and bevacizumab for the treatment of cancers was expected to enhance the efficacy of both drugs. Krasner and coworkers reported a single-arm phase II clinical trial that evaluated the efficacy of NLG 207 and bevacizumab combination treatment for recurrent ovarian cancer. The combination therapy enhanced the efficacy of NLG 207 alone. The PFS of combinatory therapy increased from 4.5 months to 6.5 months and ORR increased from 11% to 18% in comparison with NLG 207 alone [69]. NLG 207 was also combined with standard neoadjuvant chemoradiotherapy (radiotherapy + capecitabine) to treat advanced rectal cancer. The MTD of weekly dosing was identified as 15 mg/m^2^. At the biweekly dosing of MTD, 6 out of 32 (19%) patients obtained pCR (pathologic complete response). Meanwhile, 2/6 (33%) patients who received weekly MTD achieved pCR [70].

### 3.2. CD-CPT Conjugate

Shi et al. synthesized a polyprodrug with redox-responsive amphiphilic starburst diblock copolymers termed CCP(SS) (β-CD-PCPTSS-POEGMA). CCP(SS) consisting of a hydrophobic polymerized CPT prodrug (pCPT) block and a hydrophilic poly[(ethylene glycol) methyl ether methacrylate] (POEGMA) block grafted to CDs could form unimolecular micelles with an average diameter of 23.9 ± 5.3 nm. Under highly reductive conditions such as tumor cells rich in thiols, drug release from the CCP(SS) could be effectively induced upon the reductive cleavage of disulfide bond, leading to tumor-specific CPT release. CCP (SS) micelles exhibited high tumor accumulation and high-efficiency tumor growth inhibition both in vitro and in vivo. In addition, CCP (SS) micelles had low toxicity to normal cells [71]. A similar poly-irinotecan (Ir) prodrug was synthesized by the same group. Ir is a CPT analog that has been approved by FDA for clinical application. Ir was first reacted with MABHD (2-((2-hydroxyethyl) disulfanyl) ethyl methacrylate) to produce the disulfide bond-contained Ir monomer (MABHD-Ir). Subsequently, the polyprodrug CPIO (β-CD-P(Ir-co-OEGMA)) was synthesized via the facile ATRP method using MABHD-Ir and OEGMA as the comonomers and β-CD-Br as the macroinitiator. The star-like amphiphilic poly-Ir prodrug could readily self-assemble into spherical micelles with a diameter of about 41.9 nm. CPIO micelles exhibited enhanced antiproliferative effects in comparison with free Ir against Hela and MCF-7 cells and showed excellent biocompatibility on a mouse model. In addition, CPIO micelles could enter cancer cells in a time-dependent manner through the lysosomal pathway [72].

Yang and colleagues conjugated CPT to β-CD via ester linkage and click chemistry to improve the aqueous solubility of CPT. Taking advantage of the strong binding ability between adamantane and β-CD, adamantane-grafted hyaluronic acid (HA-ADA) was included in the CD cavity of CPT-CD through host–guest interactions to obtain a supramolecular nanoparticle (HACPTPs). The spherical nanoparticles were about 118 nm in size with a negatively charged surface due to the existence of negatively charged hydrophilic HA shell. The negatively charged surface is beneficial to the stability, dispersibility and biocompatibility of HACPTPs in a physiological environment [73]. The presence of HA on the surface could specifically target the HA receptor (CD44)-overexpressed malignant cancer cells. Meanwhile, hydrophobic CPT located in the core avoided the hydrolysis of the lactone ring, which could be easily hydrolyzed to inactive carboxylate form. HACPTPs could be uptaken by cancer cells via HA receptor-mediated endocytosis, and showed comparable cytotoxic effects but better safety profiles with commercial CPT [74].

Gao et al. synthesized a reduction and pH dual-responsive polyprodrug containing a hydrophilic POEGMA (poly-(ethylene glycol) methyl ether methacrylate) chain and a hydrophobic CPT prodrug and PDPA (poly[2-(diisopropylamino)ethyl methacrylate]), termed CCDO. CPT was conjugated to the copolymers via disulfide bond, which could be broken at a reduction environment such as high-level GSH in tumor cells. The presence of a DPA (2-(diisopropylamino) ethyl methacrylate) chain in the polyprodrug might be protonated in an acidic tumor microenvironment, leading to a transformation from hydrophobicity to hydrophilicity and further accelerating the drug release. CCDO was able to form stable unimolecular micelles and Dox could be encapsulated into the micelles. The Dox-loaded CCDO micelles were efficiently uptaken by cancer cells (Hela and MCF-7 cells). In addition, CCDO/Dox was more cytotoxic than CCDO, probably due to the synergistic effect of Dox and CPT, which are known to inhibit topoisomerase II and topoisomerase I, respectively [75]. Moreover, CCDO and CCDO/Dox showed good blood biocompatibility in vivo [76].

### 3.3. CD–Dox Conjugate

Liu et al. fabricated a β-CD-based star copolymer with Dox and FA coupled to HPMA ((2-hydroxypropyl) methacrylamide), which was conjugated on the lower rim of the CD cone. Meanwhile, contrast agent DOTA-Gd moiety was conjugated on the upper rim of the CD toroid cone via click reaction. The resulting (DOTA-Gd)_7_-CD-(PHPMA-FA-DOX)_14_ star copolymer consisted of 7 DOTA-Gd and 14 HPMA arms that was covalently conjugated with Dox and FA through pH-sensitive carbamate bonds and ester linkages. The presence of Dox with a drug loading content of about 14% endowed the hydrophilic CD-based star polymer with amphiphilicity. (DOTA-Gd)_7_-CD-(PHPMA-FA-DOX)_14_ could self-assemble into narrowly distributed micellar nanoparticles with a mean particle size of 26 nm. The star copolymer exhibited potent anticancer activity at a Dox concentration higher than 80.0 mg/mL. In addition, (DOTA-Gd)7-CD-(PHPMA-FA-DOX)_14_ significantly enhanced T_1_ relaxivity (r1 = 11.4 s^−1^ mM^−1^) in comparison with the small molecular counterpart alkynyl-DOTA-Gd (r1 = 3.1 s^−1^ mM^−1^) [77].

For these CD–drug conjugates, stimuli responsiveness could be easily introduced into the copolymer structures. Jia et al. synthesized a pH-sensitive star-like CPOFs via the ATRP method with OEGMA (poly(oligo-(ethylene glycol methyl ether methacrylate) and benzoaldehyde as comonomers and β-CD-21Br (heptakis [2,3,6-tri-o-(2-bromo-2-methylpropionyl]-β-cyclodextrin) as the macroinitiator. Dox was covalently linked to the aldehyde group of CPOFs polymer through pH-responsive Schiff-base bonds. The CPOF-Dox conjugate was readily to form robust unimolecular micelles with spherical shape and average diameter of 18 nm. The covalently conjugated Dox was more stable and release more controllable than the noncovalently encapsulated Dox [78]. Shi and coworkers synthesized an acid-activatable star-like poly-Dox prodrug through introduction of a hydrazone bond. The poly-Dox prodrug (Dox@CPMO) was composed of β-CD-PMGMA (poly(2-methoxy-2-oxoethyl methacrylate)) with hydrophilic POEGMA (poly[(ethylene glycol) methyl ether methacrylate] conjugated at the end of β-CD-PMGMA via ATRP reaction and Dox grafted to the chain through the hydrazone bond. The drug loading content of Dox@CPMO was extremely high (up to 53.1%). Dox@CPMO could form stable unimolecular micelles with adjustable sizes by changing the ratio of hydrophobic PDOX chains and hydrophilic POEGMA chains. Furthermore, Dox@CPMO micelles showed a higher growth inhibition effect against tumor cells but lower toxicity to normal HUVEC cells. More importantly, Dox@CPMO micelles may serve as fluorescent nanoprobes for tumor diagnosis due to the pH-sensitive fluorescence activated by the acidic tumor microenvironment [79].

To better control or adjust drug release under different stimuli conditions, dual- or multiple-responsive carriers were developed. Bai and colleagues constructed a photo and pH dual-responsive supramolecular prodrug complex consisting of β-CD-acylhydrazone-Dox and FA-modified PDMA (poly(2-(dimethylamino)ethyl methacrylate)) with azobenzene ends. The Dox-loaded supramolecular complex could further form into multicompartment self-assemblies. UV light could trigger the dissociation of β-CD/Azo, leading to the transformation of vesicles and slightly increased Dox release. The acidic environment could induce the cleavage of acylhydrazone that linked Dox and β-CD, resulting in remarkably accelerated Dox release from the nanoassemblies [80].

## 4. CD-based Copolymer

Conjugation of proper polymers onto CDs has attracted increasing attention in the field of anticancer drug delivery systems. Due to the abundant hydroxy groups, polymers or macromolecules are easily connected onto CDs. The CD-copolymers could self-assemble into core–shell nanoparticles if proper polymers or macromolecules are conjugated on CDs. Zhang and Ma developed a CD-based hydrophilic diblock copolymer, PEG-b-PCD. This copolymer contains a PEG block and a polyaspartamide block grafted with β-CDs on the side chain via EDA (ethylenediamine) linkages. Hydrophobic drugs or macromolecules can be carried in the interior lipophilic core of CDs by host–guest interaction. The resulting amphiphilic inclusion complex can spontaneously assemble into nanospheres. Additional apolar drugs can be entrapped into the hydrophobic core of nanospheres through hydrophobic interaction [81].

### 4.1. CD-Grafted Copolymer with Linear Chains

There are 21 hydroxy groups on a β-CD molecule. The modification of one hydroxy group with linear polymers may obtain the linear CD copolymers. The conjugation of CDs and polymers can be realized through different linkages. Therefore, stimuli-responsive bonds could be introduced to facilitate the release of chemotherapeutics at tumor sites. The relatively acidic environment of tumor tissues in comparison with normal tissues provides the possibility to deliver anticancer drugs through a pH-dependent manner. The introduction of acid-labile chemical bonds such as imine, hydrazone or acetal into polymer backbones or side chains renders the polymer structure to remain stable at physiological pH (7.4), but cleaves quickly at the relatively lower pH condition of the tumor microenvironment, indicating a rapid drug release [82,83,84]. Zhao MR et al. synthesized an amphiphilic mono-6-β-CD octadecylimine (6-β-CD-N-ODMA) copolymer with octadecylamine coupled on CD via imine linkage. 6-β-CD-N-ODMA can self-assemble into polymeric micelles and PTX can be encapsulated into the micelles by a dialysis method with a drug loading content of 1.97%. The PTX release from the polymeric micelles was faster in an acidic environment (pH 5.0) compared to pH 7.4, and the release profile at pH 5.0 was compatible with the Ritger–Peppas equation [85]. Excessive ROS (reactive oxygen species) are produced in cancer cells owing to mitochondrial dysfuction, abnormal metabolic process metabolism, increased cell signaling and elevated peroxisome activities [86,87]. Therefore, ROS could specifically serve as a trigger for the delivery of anticancer agents. Jia and coworkers developed ROS-responsive MPEG-CD-PHB (PCP) micelles for the codelivery of Dox and photosensitizer P18 (purpurin-18). Mono(6-amino-6-deoxy)-β-CD was conjugated with PEG-NHS to generate MPEG-CD. A borate bond was introduced into the copolymer through the grafting of PHB (4-(hydroxymethyl) phenylboronic acid pinacol ester) to accelerate the release of entrapped Dox and P18 in the presence of high content of ROS in the tumor microenvironment. The Dox- and P18-coloaded PCP micelles showed enhanced anticancer activity both in vitro and in vivo, and a synergistic effect was observed. Additionally, PCP micelles exhibited good blood compatibility and excellent biosafety in vivo [88].

Targeting ligands could be introduced into the CD-grafted linear copolymers to efficiently deliver chemotherapeutics to the tumor sites. Fan and colleagues synthesized FA-conjugated β-CD with PEG as the linker. The resulting FA-PEG-β-CD copolymer could form nanoparticles with a size of about 20–80 nm. Dox was loaded in the particles with a loading content of 11.9% and encapsulation efficiency of 95.2%. Dox-loaded nanoparticles released faster in acidic conditions than pH 7.2, probably due to the presence of a Schiff base bond between PEG and β-CD and the more hydrophilic nature of Dox in a low-pH environment [89]. Hyun et al. synthesized a similar FA-decorated CD copolymer with PEG as the linker, termed as CDPF. Unlike Fan’s copolymer linked by a Schiff base, mono-6-amino-β-CD was conjugated to PEG with an amide bond in CDPF. Dox-loaded CDPF exhibited a particle size distribution in the range of 38 nm to 52 nm. CDPF-Dox showed 2.9-fold and 7.7-fold tumor growth inhibition enhancement in MCF-7 cells and MCF-7-bearing tumor mice compared to free Dox, respectively. In addition, CDPF-Dox reduced the cardiotoxicity and systemic toxicity of Dox [90]. LA (lactobionic acid) is an attractive hepatocyte-target ligand that can be used for liver cancer drug delivery [91]. Yang and colleagues designed an LA-modified CD-PEG copolymer (LA-PEG-β-CD) by coupling β-CD and LA to a PEG chain with alkynation and click reaction. Dox was modified with benzimidazole through a hydrazone bond to produce a prodrug as an object–chain segment. LA-PEG-β-CD and BM-Dox could self-assemble into nanoscale particles via host–guest reaction. At acidic tumor sites and the intracellular acidic environment, the hydrazone bond could be easily cleaved, resulting in the accelerated release of Dox. In addition, the protonation of benzimidazole at acidic conditions could boost the decomposition of the host–guest complex and further facilitate the fast release of Dox. In vitro anticancer studies showed that LA modification enhanced the cytotoxicity of the PEG-CD/BM-Dox prodrug complexes [92].

### 4.2. CD-Based Star-Shaped Copolymers

**Figure 4:**
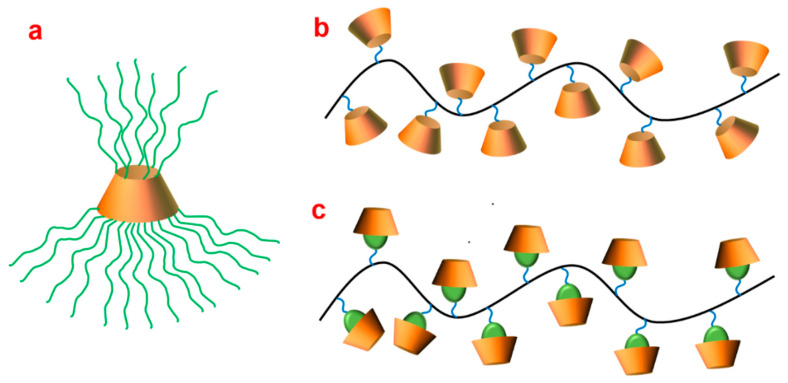
Schematic illustration of star-shaped copolymers (**a**), polymers with pendant CD (**b**) and polymers with pendant guest components (**c**).

Unlike linear polymers, star-shaped polymers consist of a core structure, and multiple polymer arms are grafted to the core and radiate outwards (Figure 4a) [93]. Over the past few decades, star-shaped polymers have been exploited to improve the water solubility of drugs and enhance the therapeutic efficacy and targeted delivery of drugs to specific disease tissues [94]. It is reported that polymeric micelles formed by conventional linear block copolymers have low stability due to high dilution in the circulation system. The stability issue of polymeric micelles can be addressed by star-shaped copolymers owing to their ability to form more stabilized unimolecular micelles. Cyclodextrin with abundant active hydroxy groups can be employed as a core molecule for synthesizing star-shaped polymers. Apart from the unique characteristics of star polymers, CD-based star-shaped polymers automatically have the advantages of entrapping hydrophobic drugs into their lipophilic cavity via host–guest interaction [95,96]. In addition, the grafted polymer arms provide the possibility to introduce segmented blocks that are sensitive to various stimuli such as pH, ROS, temperature or redox. These stimuli-responsive polymers could release drugs precisely at tumor sites, and thus improve therapeutic efficacy. Therefore, CD-based star-shaped polymers are of great interest for applications in the biomedical field.

#### 4.2.1. Stimuli-Responsive Star-Shaped Copolymers

##### Oxidation- and Reduction-Responsive Copolymers

Differences between tumor and normal tissues promote the development of stimuli-sensitive delivery system. For instance, the overproduction of both ROS (reactive oxygen species) and GSH (glutathione) in tumor cells leads to the development of an on-demand tumor-specific stimuli-sensitive delivery system [97,98]. Zuo et al. reported a group of oxidation-responsive star-like amphiphilic copolymers with β-CD-conjugated POEGMA (poly(oligo ethylene glycol) methacrylates) as the host molecule and Fc(ferrocene)-modified star-shaped lipophilic PCL (poly(ε-caprolactone)) block as the guest counterpart. The star-shaped block copolymers with 9, 12 and 18 arms were fabricated by mixed star PCL-Fc with 3-arm-β-CD POEGMA at molar ratios varying from 1:3, 1:4 to 1:6. The polymeric micelles formed from 12-arm star-like copolymers showed the highest CAC (critical aggregation concentration) value (0.506 mg/mL) among the star-shaped copolymers. The relatively higher CAC value may contribute to a superior stability against dilution in comparison with other copolymers [99]. DOX was encapsulated into the micellar core of PCL-Fc/β-CD-POEGMA micelles and the 12-arm star-shaped micelle showed a drug loading content of 6.49% and encapsulation efficiency of 42.7%. The excess amount of ROS (reactive oxygen species) produced in tumor tissues was ready to oxidize the reduced Fc to Fc+, which weakly binds to CD, facilitating the dissociation of CD-Fc complexes [100,101]. In vitro release studies showed that the presence of oxidative medium significantly accelerated the release of DOX from the drug-loaded PCL-Fc/β-CD-POEGMA micelles [102].

Compared with normal cells, tumor cells have a 4-fold higher GSH concentration. In addition, the intracellular concentration (2–10 mM) of GSH is about ~1000 times higher than the extracellular environment (2–20 μM). Therefore, differences between tumor cells and normal cells as well as intracellular and extracellular environments facilitate the development of redox-responsive drug delivery systems [103,104]. Hu’s group synthesized a panel of redox-responsive supramolecular pseudoblock polycations (CD-SS-pDM/Ad-pPEGs), in which pDM (poly (2-dimethyl amino)ethyl methacrylate) was conjugated on β-CD core via a disulfide bond, and the resulting star-like CD-SS-pDM was complexed with adamantine-ended pEGEEMA (poly(poly(ethylene glycol)ethyl ether methacrylate)) through host–guest interaction. The pseudoblock carriers CD-SS-pDM/Ad-pPEGs showed a comparable pDNA condensing effect with corresponding CD-SS-pDMs. CD-SS-pDMs/Ad-pPEG/pDNA complexes showed rapid pDNA release under reducible conditions, which may facilitate intracellular pDNA release and thus regulate gene expression. Interestingly, CD-SS-pDMs/Ad-pPEG exhibited decreased toxicity, enhanced gene transfection efficiencies and cellular internalization compared to the corresponding CD-SS-pDMs at various N/P ratios. This might be due to the introduction of pEGEEMA to the polycations [105,106]. Furthermore, in vivo studies showed that CD-SS-pDMs/Ad-pPEG could deliver the p53 gene more effectively into tumor tissues and more potently suppress tumor growth in mice [107].

##### pH-Responsive Copolymers

Lv and colleagues synthesized about 60 types of β-CD-based amphiphilic star-like copolymers. P(AA-co-MMA)-PVP (poly (acrylicacid)-co-poly(methyl methacrylate)-poly (vinyl pyrrolidone)) or P(AA-co-MMA-co-A1-b-CD)-PVP (poly (acrylic acid)-co-poly(methyl methacrylate)-co-poly(monoacylated-b-CD)-poly (vinyl pyrrolidone)) blocks were conjugated to the primary hydroxy groups of 2, 3 and 6 positions of β-CD. VP (Vinpocetine) was loaded in the optimal β-CD-P4 micelles with an average size, loading content and entrapment efficiency of 67.4 nm, 21.44 ± 0.14% and 49.05 ± 0.36%, respectively. The release rates of VP-loaded β-CD-P4 micelles in acidic medium were much faster than in physiological conditions. The pH sensitivity of the β-CD-P4 micelles may be due to the PAA blocks with a pKa of 4.5 and a much faster hydrolysis of cyclodextrin under an acidic environment. Interestingly, VP-loaded β-CD-P4 micelles were more likely to be distributed in lung tissues with targeting efficiency 8.98-fold greater than VP injection [108]. The amphiphilic copolymer was similar to alveolar surfactants, facilitating the alveoli cell absorption and thus enhancing lung targetability [109,110]. Xu Z et al. developed a star-shaped copolymer consisting of a β-CD core with 21-arm PLA-b-PEG (poly(lactic acid))-b-poly(ethylene glycol) conjugated on the free hydroxy groups. Monodispersed, spherical and stable unimolecular micelles were formed with average diameters of 10~20 nm. Dox was loaded in the micelles and exhibited a pH-controlled release, probably owing to an elevated solubility of Dox in acidic medium and accelerated degradation of PLA at pH 5.0 [111].

**Figure 5:**
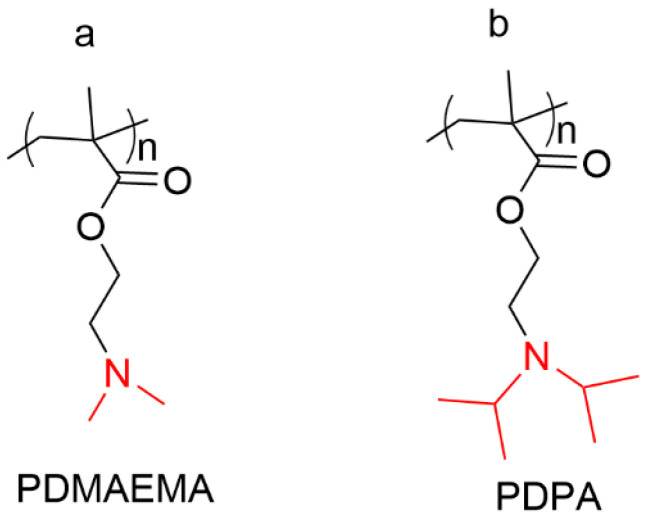
Chemical structure of PDMEMA (**a**) and PDPA (**b**).

The introduction of PEMTA [(poly(ethyl methacrylate) with tertiary amine group) at the other end of the ethyl group is a useful approach to acquire pH sensitivity for star-like copolymers. PEMTA segments retain hydrophobicity at basic or neutral environments. Under acidic conditions, the tertiary amine groups of PEMTA can be protonated and turned hydrophilic, leading to the swelling of the hydrophobic domain and further accelerating the release of the entrapped drug [112,113]. PDMAEMA (Poly(2-(dimethylamino) ethyl methacrylate)) (Figure 5a) and PEG were grafted to β-CD to produce a star-like amphiphilic copolymer. The star-like copolymer was able to form micelles with hydrophobic guest molecules such as the anticancer drug Dox. Dox could be released in response to the acidic endosomal-pH after being uptaken by Hela cells via a nonspecific endocytosis approach. In addition, the Dox-loaded micelles were more potent than free Dox against cancer cells at high concentrations [114]. The Dox-loaded star-like copolymer micelles could also suppress the growth of tumors in cervical tumor-bearing mice with a higher growth inhibition rate and lower side effect compared to free Dox [115]. Lin et al. developed dual-functional unimolecular micelles through the grafting of PDMAEMA as pH-sensitive and a cationic block on β-CD-based star-shaped copolymers for the codelivery of imiquimod and plasmid DNA to dendritic cells. The release of imiquimod could be accelerated at the mildly acidic endolysosomal pH (5.0), where its target, TLR7, is located [116]. The star polymer-formed unimolecular micelles were capable of condensing plasmid DNA and transfecting 6~15% dendritic cells. The gene transfection efficiency of the unimolecular micelles did not alter with codelivery of imiquimod [117]. PDPA (poly(2-(diisopropylamino) ethylmethacrylate) was also utilized as a pH-responsive block for the construction of CD-based star-like copolymers. An amphiphilic diblock star-shaped copolymer conjugated with PDPA-b-POEGMA (poly[(ethylene glycol) methyl ether methacrylate])(Figure 5b) on the hydroxy groups of β-CD via ATRP was developed by Shi and coworkers for the delivery of anticancer drugs. The amphiphilic β-CD-PDPA-POEGMA copolymer (CPO) could self-assemble into stable spherical unimolecular micelles with average diameters of ~20 nm. Additionally, the particle size could be regulated by varying the lengths of the POEGMA blocks and PDPA blocks. Dox could be encapsulated in the micelles with high drug loading content up to 30%. The Dox-loaded CPO micelles exhibited high selective cytotoxicity against cancer cells (MCF-7 and Hela cells) and weak toxicity for normal cells (L929 cells) [118]. This β-CD-PDPA-POEGMA star-like copolymer can also coencapsulate the anticancer drug DOX and photothermal agent BBT-2FT (benzo[1,2-c;4,5-c’] bis [1,2,5] thiadiazole-4,7-bis(9,9-dioctyl-9H-fluoren-2-yl) thiophene) with high drug loading content (8.87% for DOX; 4.02% for BBT-2FT). The Dox- and BBT-2FT-loaded unimolecular micelles exhibited increased in vitro release rates under acidic conditions and irradiation, and showed elevated therapeutic efficacy against cancer cells [119].

##### Thermoresponsive Polymers

Thermoresponsive polymers, exhibiting a reversible phase transition to temperature, have shown great potential in biomedical areas. Thermoresponsive polymers can be classified into LCST (lower critical solution temperature) types and UCST (upper critical solution temperature) types [120,121]. Among them, PNIPAAm (Poly (N-isopropylacrylamide))-based polymers are one of the most widely studied thermoresponsive polymers owing to their intense volume change with a lower LCST (32 °C). PNIPAAm is water-soluble below its LCST due to the interactions broken between amide groups and expansion of its polymer chains. On the contrary, PNIPAAm become water insoluble above LCST due to the collapsing of polymer chains caused by water repelling from the polymer network [122,123,124]. Song X and colleagues reported a thermosensitive star-like copolymer comprising of a β-CD core grafted with PNIPAAm. PTX was loaded on the β-CD-(PNIPAAm)4 four-arm star copolymer via host–guest interaction. The β-CD-(PNIPAAm)4/PTX inclusion complex greatly improved the water solubility of PTX at room temperature (25 °C) due to the hydrophilic nature of PNIPAAm below its LCST. When the temperature increased to 37 °C above its LCST, PNIPAAm became hydrophobic due to the phase transition and triggered the formation of nanoparticles, which facilitated the cellular uptake. Particularly, the β-CD-(PNIPAAm)4/PTX inclusion complex showed higher antitumor effects compared with Taxol in both resistant and sensitive prostate cell lines at 37 °C [125]. β-CD-(PNIPAAm) was also able to form an inclusion complex with guest polymers. Due to their high association constant of more than 104 when complexing with β-CD, adamantane- and adamantyl-based polymers are able to form stable inclusion complexes with β-CDs [126]. Adamantyl-conjugated PEG (Ad-PEG) moiety was complexed with β-CD-(PNIPAAm) to form a pseudoblock copolymer β-CD-(N21)4/Ad-PEG. At body temperature, the pseudoblock star-shaped copolymer can form micelles capable of loading Dox with a loading content of 6%. The Dox-loaded β-CD-(N21)4/Ad-PEG micelles were more potent than free Dox against cancer cells. In addition, unlike free Dox, the Dox-loaded micelles showed high cytotoxicity in resistant AT3B-1 (MDR+) cells. This enhanced therapeutic efficacy against resistant cells may presumably be due to micelles escaping the recognition and resistant mechanisms of the MDR+ cells [127].

To improve the stability of β-CD-(PNIPAAm) self-assemblies, biocompatible PEG can be imbedded in the star-shaped copolymers. Fan X et al. synthesized a star-like copolymer, β-CD-v-(PEG-β-PNIPAAm)7, with V-shaped arms to the thermosensitive delivery of Dox or PTX through host–guest interaction to cancer cells. At body temperature, the phase transition of PNIPAAm in the copolymers was able to induce the polymer–drug inclusion complex from nanoassemblies, which could significantly enhance the cellular uptake and intracellular drug concentration, and further led to the inhibition of MDR-1 protein-associated drug resistance. Both Dox- and PTX-loaded β-CD-v-(PEG-β-PNIPAAm)7 nanoparticles could efficiently suppress the growth of drug-resistant cancer cells. Furthermore, PTX-loaded assemblies showed significantly higher growth inhibition without a negative impact on the normal tissues in the mice model bearing resistant HepG2/MDR1 tumor xenografts compared to the commercially available Taxol. This may be probably due to the increased retention of PTX-loaded supramolecular nanoassemblies in the resistant tumor tissues [128].

##### Dual-Responsive Copolymers

By introducing stimulus groups into the polymers, the polymeric carriers are able to rapidly release anticancer drugs into tumor tissues while slowly releasing them into normal tissues, leading to enhanced therapeutic effects and reduced side effects. To improve further drug efficacy, double or multiple stimuli-responsive units could be simultaneously introduced into the delivery systems to achieve synergistic release effects [129]. Adeli et al. developed a thermo/pH dual-sensitive micelle based on β-CD-grafted PMAA-b-PNIPAM (poly(methacrylic acid)-block-poly(N-isopropylacrylamide)) star-like copolymers as the host molecule and a brush-like copolymer with benzimidazole pendent groups as the guest molecule. The host–guest systems can self-assemble into micelles with mean particle sizes of about 80 nm due to their amphiphilic nature. The pH responsiveness of the micelles was obtained attributing to the presence of PMAA segments in the host copolymer chains and the benzimidazole in the guest copolymer chains provided. Meanwhile, the introduction of PNIPAM units into the star-shaped CD-grafted copolymer provided thermoresponsiveness to the micelles. Dox was loaded in the micelle with drug loading content of 9.73% and encapsulation efficiency as high as 97.3%. Micelle-loaded Dox exhibited enhanced tumor growth inhibition efficacy compared to free Dox against MCF-7 cell line [130]. Zhou and coworkers synthesized a star-shaped thermoresponsive copolymer β-CD-PNIPAM as the host and pH-responsive BM-PCL (benzimidazole-terminated poly(ε-caprolactone)) as the guest. Supramolecular spherical micelles were formed with β-CD/BM-PCL as the core and PNIPAM as the shell. Dox was encapsulated in the supramolecular micelles with a drug loading content as high as up to 36.38%. The main reason for the high loading content might be that both the lipophilic micelle cores and β-CD cavities can entrap drugs. The temperature and pH remarkably affected the release of Dox. When pH decreased from 7.0 to 5.2 and temperature increased from 25 to 37 °C, the release rate of Dox from the dual-responsive supramolecular micelles was significantly accelerated. In addition, the tumor cell growth inhibition of Dox-loaded micelles was greater than free Dox [131]. To obtain efficient intracellular drug release, Zhou and colleagues developed a dual pH-sensitive supramolecular delivery system. PDMAEMA grafted on β-CDs and benzimidazole embedded into the CD cavities jointly contributed to their pH sensitivity. Dox could be encapsulated in the dual-pH-responsive supramolecular micelles with high drug loading content (more than 40%). The resulting Dox-loaded micelles exhibited higher tumor growth inhibition than free Dox. Interestingly, Dox release could be regulated not only by altering the pH, but also by changing the temperature. This may be due to the destruction of micellar stability caused by the transformation of PDMAEMA chains from an expanding state to a compact state as the temperature increases [132].

#### 4.2.2. Tumor-Targeted Star-Shaped Copolymers

An ideal drug delivery system should remain stable in the circulation system and be able to deliver therapeutic agents to the desired disease tissue. To achieve the specific accumulation at tumor sites, an active targeting approach with a targeting moiety conjugated/decorated to the nanoparticle surface seems be an attractive strategy for cancer therapy [133]. Folic acid (FA) receptors are overexpressed on the surface of 40% solid tumors including ovarian, cervical, epithelial, breast, kidney and colorectal tumors [134,135]. The integrating of active targeting and stimuli-responsive characteristics is able to realize rapid intracellular drug release after receptor-mediated endocytosis in the targeted cells. Hong et al. developed a nano-drug system (FA-Cur-NPs) with β-CD, and ε-CL (ε-caprolactone)-modified β-CD as carriers for curcumin. The near-spherical nanosized FA-Cur-NPs were about 150 nm, with a high drug loading capacity (>20%). In acidic medium, FA-Cur-NPs exhibited a 3-fold faster release at pH 6.4 (mimicking the tumor microenvironment) than at pH 7.4, which might be owing to the accelerated degradation of ester bonds in acidic conditions. FA-Cur-NPs showed a higher intracellular uptake than Cur-NPs (without FA) and enhanced tumor growth inhibition both in vitro and in vivo; this might be ascribed to the folate receptor-mediated endocytosis [136]. Sun and colleagues developed a ginsenoside Rg3 (Rg3) and quercetin (QTN) coloaded formulation based on star-shaped amphiphilic β-CD and folate-modified DSPE-PEG as the carriers. The CD-PEG-FA.Rg3.QTN coformulation showed higher drug release rates at acidic PBS than neutral PBS; the mechanism might be attributed to the protonation of amine groups at acidic environment. By codelivering QTN, the ICD (immunogenic cell death) efficacy of Rg3 was remarkably boosted because QTN could induce ROS (reactive oxygen species). In orthotopic colorectal tumor mouse model, the FA targeted coformulation reversed the immunosuppressive feature of TME (tumor microenvironment) and greatly prolonged the survival of cancerous mice in combination with PD-L1 [137,138].

The introduction of the disulfide bond could trigger rapid drug release upon elevated intracellular GSH levels in tumor cells. An amphiphilic star-like copolymer, β-CD-g-PCL-SS-PEG-FA, was developed by Li and coworkers through the conjugation of PCL-grafted β-CD and PEG-FA via disulfide linkage. Dox could be complexed with β-CD-g-PCL-SS-PEG-FA to form unimolecular micelles with a loading rate of 8.1% and a particle size of 31.5 nm. The encapsulated Dox in the micelles could be released in a GSH-dependent manner. The cellular uptake of β-CD-g-PCL-SS-PEG-FA/DOX in HeLa/MDR1 drug-resistant cells was significantly enhanced compared with free Dox and Dox-loaded micelles without FA. In addition, the inhibitory effect of β-CD-g-PCL-SS-PEG-FA/DOX on multidrug-resistant Hela cells was remarkably increased in comparison with free DOX. The enhanced intracellular uptake and tumor inhibition rate might be due to the highly expressed FR (folic acid receptors) promoting the uptake of FA modified micelles and the high intracellular GSH concentration causing rapid release of Dox [139]. Cationic star-shaped copolymers can also be modified with FA through bioreducible disulfide linkage to possess the ability to target delivery of gene agents to tumor cells. For efficient folate-targeted delivery, an dense enough level of FRs on the membrane surface of tumor cells is a prior condition for FR-mediated endocytosis [140]. Therefore, the continuous recovery and recycling of FRs after internalization to sustain sufficient FRs on the cell membrane is of great importance. The high-concentration intracellular GSH leads to the cleavage of the disulfide linker in the copolymer, leading to FR recycling and recovery onto the membrane surface to facilitate continuous FR-mediated endocytosis of gene agents to obtain enhanced gene expression [141]. Zhao et al. developed a star-like cationic copolymer consisting of multiple OEI (oligoethylenimine) arms grafted γ-CD with FA conjugated through bioreducible disulfide linkage. PTX was encapsulated in the hydrophobic cavity of γ-CD with a corresponding loading level of 10.4%, and further condensed with the p53 gene to form nanoparticulate polyplexes. The FA-targeted γ-CD-OEI-SS-FA/PTX complex could significantly enhance gene transfection efficiency in the FR-positive KB cells. The presence of the disulfide bond enabled enhancement of the FR recovery and recycling from cytosol after particle internalization, resulting in further enhancement of gene transfection. Interestingly, codelivery of PTX also enhanced the gene transfection efficiency. The wild-type p53 gene could efficiently be delivered to FR-positive KB cells and effectively induce tumor cell apoptosis [142].

**Figure 6:**
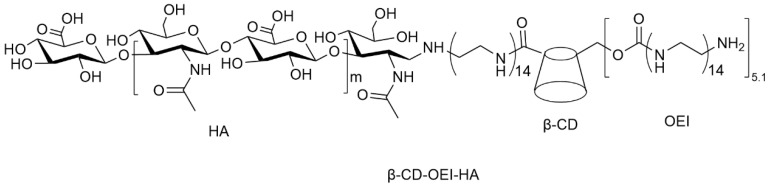
Chemical structure of β-CD-OEI-HA.

CD 44, a nonkinase transmembrane glycoprotein, is a specific target for hyaluronic acid (HA). High expression of CD44 is dramatically ascribed to the proliferation, metastasis, invasion and migration of tumor cells. Yin and colleagues constructed a CD44-targeted star-shaped cationic copolymer for gene delivery. Low-molecular-weight HA was conjugated with cationic β-CD-OEI star polymer to produce β-CD-OEI-HA copolymer (Figure 6). β-CD-OEI-HA copolymer could efficiently condense pDNA to form nanoparticles with diameters of 100~200 nm at N/P ratios of 8 or higher. The negatively charged HA conjugated on β-CD-OEI may neutralize the cationic OEI segment, thus reducing the cytotoxicity of β-CD-OEI-HA/pDNA complexes. In addition, β-CD-OEI-HA exhibited significantly higher gene transfection efficiency in CD44 overexpressed MDA-MB-231 cells in comparison with PEI (25 kDa) and β-CD-OEI due to the active targeting of HA. More importantly, the wild-type p53 gene could be effectively delivered to CD44-positive MDA-MB-231 cells, leading to enhancement of cell apoptosis and increased cell growth inhibition [143].

### 4.3. CDs Grafted on Polymer Chains or Complexed on Polymer Side Chains with Brush-like Structures

Unlike star-shaped copolymers with polymer branches linked to CD core, brush-like copolymers consisting of polymer with CDs grafted onto the backbone/or complexing guest segment are linked on the linear chain (Figure 4b,c). There should be abundant active functional groups in the polymer suitable for CDs or other guest molecule conjugation. Some biocompatible and biodegradable polymers, including polysaccharide and polypeptides, have been used to graft CDs on their side chains.

#### 4.3.1. Brush-like Copolymers as Hosts (Polymers with Pendant CDs)

Chitosan has been widely used in drug delivery systems due to its low cost, biodegradability, biocompatibility and nontoxicity. As a linear polysaccharide consisting of randomly distributed D-glucosamine (deacetylated unit) and N-acetyl-D-glucosamine (acetylated unit) via β-(1→4) glycosidic linkage, there are sufficient active hydroxyl groups and amino groups, which give opportunities to conjugate with CDs [144]. Gao et al. synthesized a β-CD-grafted chitosan copolymer by conjugating the hydroxyl group of β-CD at the C-6 position and the amino group of chitosan. The resulting CS-g-CD (chitosan-grafted cyclodextrin) was complexed with BM-PCL (benzimidazole-terminated poly(ε-caprolactone)) via host–guest interaction to form complex micelles. Due to the existence of benzimidazole, the complex micelles exhibited pH sensitivity because the hydrophobic BM molecule could be protonated in an acidic environment, leading to the decomposition of the host–guest complex. Dox could be loaded into the core–shell assemblies with a high drug loading content up to 36.38%. Interestingly, the Dox-loaded complex micelles exhibited a higher drug release rate at 37 °C than at 25 °C; this might be due to a weakened host–guest interaction as the temperature increased [145,146]. CD-grafted chitosan can also be used to deliver gene agents. Ping et al. conjugated CD to the chitosan backbone through a low-molecular-weight PEI chain to produce two polycations named CPC1 and CPC2. Gene agents such as pDNA (plasmid DNA) and siRNA (small interfering RNA) could be effectively condensed by the chitosan backbone together with PEI. Meanwhile, hydrophobic molecules such as Ad-PEG (adamantyl-modified PEG) could be incorporated into the cavity of CD via host–guest interaction. CPC2/DNA complexes showed higher transfection efficiency than commercially available PEI, and CPC2/siRNA exhibited a superior knockdown effect in HEK293 and L929 cell lines. Interestingly, after complexing with Ad-PEG, the transfection efficiency of CPC2/pDNA decreased while the silencing effect of CPC2/siRNA increased. The possible reason might be associated with the destabilization of PEG-mediated complex in cytosol, facilitating the release of gene agents. The disassembled siRNA could be included into RNA for silencing effects, while the function of released DNA might be retarded due to cytosolic nuclease-induced degradation before entering nucleus [147].

As a high-molecular linear anionic glycosaminoglycan, hyaluronic acid (HA) composed of repeating disaccharide units is suitable for chemical modification. HA plays a pivotal role in biological functions, including cell proliferation, cell adhesion, stabilization and organization of the extracellular matrix. In addition, HA can specifically bind to CD44 with high binding affinity (Kd ≈ 10^−12^ M), a cell surface receptor overexpressed on a variety of cancer cells [148,149]. Therefore, HA is an attractive targeting agent for anticancer delivery systems. CDs can be covalently grafted onto the backbone of HA to achieve active targeting properties. Yang et al. conjugated β-CDs onto the HA backbone by ethanediamine (EDA) linkage via amide condensation reaction. A platinum prodrug, adamplatin (adamantylamine complex), was included in the cavity of β-CDs through host–guest recognition. The HACD/adamplatin could form nanosized assemblies (HAP) consisting of a hydrophobic amamplatin prodrug core and a hydrophilic CD/HA shell. In vitro and in vivo studies showed that it was biocompatible and biodegradable. In addition, HAP exhibited significantly a higher tumor growth inhibition effect and lower toxicity than cisplatin in HA-receptor-positive cancer cells and BALB/c nude mice bearing SKOV-3 cancer cells. This might be due to the HA-receptor targeting enhancing the accumulation of HAP in tumor cells and decreasing the drug accumulation in normal tissues [150]. This EDA-linked HACD was also used to complex curcumin–oxoplatin drug conjugate (Cur-Pt) and further self-assembled to nanospherical particles (HCPNs) with a mean diameter of 114 nm. Interestingly, HCPNs exhibited pH-sensitive release features in acidic environment. The secondary amine nitrogen of β-CD-EDA was likely to be partially protonated at acidic conditions, leading to unstable assemblies and accelerated drug release. HCPNs showed good biocompatibility with low toxicity to normal cells, and exhibited better anticancer activity than free Cur, cisplatin and Cur/cisplatin mixture against CD44 overexpressed PC-3 cells. Furthermore, Cur and cisplatin showed synergistic effects with CI (combination index) values less than 1 [151].

Dextran is a neutral glucan consisting of many active hydroxyl groups [152]. Yuan et al. grafted β-CD onto the main chain of dextran through EDA linkage to produce the hydrophilic Dex-CD. Fc-terminated PCL was included in the hydrophobic cavity of β-CD through host–guest interaction, and further formed nanosized spherical micelles with a narrow size distribution. The existence of Fc groups rendered the micelles capable of disassembling to external voltage. Fc could be oxidized to Fc+ under positive charge, resulting in a weakened binding between β-CD and Fc and causing the dissociation of the inclusion complex, while under negative charge, Fc+ could be reduced back to Fc, facilitating the Fc/β-CD complexing [153]. Xu and colleagues synthesized Dex-β-CD copolymer by grafting EDA-modified β-CD on the benzaldehyde-modified Dextran via Schiff base reaction. Fc-terminated PLA (poly(lactide)) could be included into the apolar cavity of β-CD to form the noncovalently grafted amphiphilic copolymer, termed as Dex-β-CD/Fc-PLA. Neutral hydrophobic Fc could be oxidized to cationic ferricenium [154,155]. Therefore, both voltage and ROS could trigger the exclusion of CD-complexed Fc. In addition, the Schiff base reaction resulting in benzoic imine linkage endowed pH responsiveness to the Dex-β-CD copolymer. With the hydrophilic Dex-β-CD and hydrophobic PLA, Dex-β-CD/FC-PLA was able to self-assemble into spherical-shaped triple stimuli-responsive nanoparticles. Dox was encapsulated into the nanoparticles with loading content of 13.5%. The Dox-loaded nanoparticles exhibited negligible toxicity to normal HUVEC cells and enhanced the cytotoxicity of Dox against A549 cells [156].

Polypeptides with active functional groups can also be utilized as the linear backbone to graft CDs. Zhang and Ma synthesized a PEG and polyaspartamide block copolymer containing β-CD grafted to the carboxyl group (PEG-b-PCD) via EDA (ethylenediamine) linkage. Hydrophobic drugs could be incorporated into the apolar cavity of CDs and further form core–shell nanoassemblies with a mean diameter less than 30 nm [81]. Xiong and coworkers conjugated β-CD to a poly-L-lysine chain through a Schiff base reaction to produce a PLCD copolymer. Dox could be included into the CD cavity in PLCD with loading content of 11.0%. The Dox-loaded PLCD was able to condense oligo RNA via electrostatic interaction to form gene and chemotherapeutic agent coloaded supramolecular nanoparticles. Negatively charged HA was further coated on the surface of the nanoparticle to obtain active tumor targetability. The HA-coated nanoparticle was spherical in shape with a mean particle size of 195.6 nm. Dox and oligoRNA could be effectively delivered into CD44 receptor-positive hepatocellular carcinoma cells by the HA-coated PLCD nanoparticle system. Furthermore, in mice bearing MHCC-97h cancer cells, HA-coated PLCD nanoparticles exhibited strong tumor accumulation ability [157]. Du and colleagues developed a β-CD grafted diblock copolymer termed mPEG-PLG(CD). Amine terminated mPEG initiated the ring-opening polymerization of BLG-NCA (γ-benzyl-L-glutamate-N-carboxyanhydride) to give the diblock copolymer mPEG-PBLG. After removal of the protection group on the side chains of PBLG, β-CD-EDA (mono-6-amine-β-cyclodextrin) units were conjugated to the carboxyl groups of the diblock copolymer to afford mPEG-PLG(CD). CPT (camptothecin) was entrapped in the hydrophobic interior of CD molecules to give the supramolecular inclusion complex mPEG-PLG(CPT@CD). The supramolecular complexes were able to self-assemble into nanospheres with a mean particle size of 98 nm. The stability of the CPT active lactone form was significantly enhanced due to the protection effects of the nanoparticles and the β-CD exterior surface. The in vitro cytotoxicity of mPEG-PLG(CPT@CD) showed a dose-dependent manner against MCF-7 cell lines [158].

Except for polysaccharides and polypeptides, other polymers can also be utilized to conjugate CDs on their side chains. Ren and colleagues developed a CD-based copolymer named PnvpCD by radical copolymerization of AEMACD (mono-6-deoxy-6-(methacrylate ethylamino)-b-cyclodextrin) and NVP (N-vinyl-2-pyrrolidone). PnvpCD can incorporated adamantane-terminated PCL to form micelles with a unique multicore structure. Unlike normal core–shell structures, the PCLAD domains are scattered in the micelles with a core size in the range of tens of nanometers [159]. Gao et al. constructed a PEG-containing poly-β-CD diblock copolymer synthesized by ATRP. Hydrophobic BM-PCL (benzimidazole end-capped poly (ε-caprolactone)) was imbedded into the cavity of β-CD to form complex micelles with a core–shell structure. Dox was loaded into the spherical particles with a high loading efficiency of up to 37.10%. The extremely high loading capacity of the complex micelles might be due to the apolar cavity of β-CD and the hydrophobic core providing sufficient lipophlic space for drug encapsulation. Additionally, hydrogen bonds formed between hydroxy groups of CD and polar functional groups of Dox may also possibly be ascribed to the high drug loading capacity. The release of Dox from the micelles exhibited a pH-sensitive behavior. The possible reason for the acidic release profile might be the protonation of BM groups in acidic conditions, resulting in the dissociation of BM-PCL from the CD cavity [160]. As an amino-rich cationic polyelectrolyte, PEI (Poly(ethylenimine)) is also capable of grafting CDs. Fan et al. grafted β-CD onto PEI via CDI activation as the host component to incorporate Ad-Dox (adamantane doxorubicin conjugate) through host–guest interaction. The TRAIL-encoded gene pTRAIL, as a therapeutic gene, could be loaded onto the PEI-CD/Ad-Dox. The Dox and gene codelivery system produced synergistical anticancer effects. In mice bearing SKOV-3 ovarian cancer cells, the Dox and gene codelivery system potently inhibited the tumor growth and dramatically prolonged the survival time. The enhanced anticancer activity may be due to the simultaneous and sustained release of the drug and gene in situ and apoptosis synergistically induced by Dox and TRAIL [161].

Rahmani and coworkers developed a pH-sensitive copolymer β-CD-g-PMA-co-PLGA (β-Cyclodextrin grafted poly maleate-co-poly (lactide-co-glycolide)). This copolymer can form micelles with a mean particle size of 34.5 nm and a low CMC (critical micelle concentration) of 0.1 µg/mL. Dox and conferone (Conf) were coloaded in β-CD-g-PMA-co-PLGA micelles with a high drug loading efficiency of up to 98%. The core of the micelle, the β-CD cavity and the presence of the electrostatic binding site (-COO-) of the PMA section may be responsible for the high loading efficiency. Dox released from the micelle showed a pH-dependent mode, with a more dominant release in acidic conditions (pH 5.5) compared to physiological conditions (pH 7.4). The reason for the pH-responsive release behavior of Dox was the electrostatic interaction between the carboxylate group of the poly maleate section and the amine group of Dox dissociated in acidic pH where the carboxylate group transferred to the carboxyl acid group (-COOH). In vitro anticancer activity against MDA-MB-231 cells showed that the Dox and Conf coloaded micelles exhibited synergistic effects with the lowest IC_50_ values in comparison with free Dox or single drug-loaded micelles. More than 98% of cells showed apoptosis after Dox and Conf coloaded micelle treatment at a dose of IC_50_ (0.259 μg/mL). The intrinsic mitochondrial apoptosis pathway (p53, p27, Bcl-2/Bax, cleave-caspase-9, cleaved caspase-7 and cleaved caspase 3) may account for the Dox and Conf coloaded micelle-induced apoptosis [162].

#### 4.3.2. Brush-like Copolymers as Guests (Polymers with Pendant Guest Components)

Adamantane methylamine was conjugated onto the hyaluronic acid backbone to produce a pendant polymer system. Adamantane on the side chains of copolymers was able to interact with cationic β-CD (CD-PEI) to form stable complexes via host/guest recognition. Negatively charged nucleic acid could be condensed with the resulting HA-Ad:CD-PEI^+^ complex to form HA-Ad:CD-PEI^+^:pDNA. This HA-based transfection system dramatically enhanced the cellular uptake and transfection efficiency in CD44-overexpressed Hela cells [163]. Additionally, microfluidic technology was used to assemble HA-Ad:CD-PEI:pDNA nanoparticles with smaller sizes, lower polydispersity and lower zeta potential. The pDNA-loaded nanocomplexes formed by flow mixing strategy were more susceptible to disassembly at higher flow rates, avoiding any chemical changes (pH, enzyme responsive linkages, etc.) to the carrier materials that were used to realize gene cargo disassembly from transfection complexes after cell internalization [164]. A stable complex with Kc > 10^4^ M^−1^ could also be formed between HA-Ad and M-β-CD. The HA-Ad/M-β-CD complex was able to self-assemble into negatively charged nanoparticles with diameter of 140 nm. Interestingly, M-β-CD was not only a guest molecule, but also an anticancer agent that can induce apoptosis in tumor cells [165]. After complexation with HA-Ad, HA-Ad/M-β-CD exhibited a more potent anticancer effect against HCT 116 cells (CD44-positive), mainly due to the CD44 receptor-mediated endocytosis [166]. To further enhance the anticancer effect of M-β-CD, Elamin and coworkers constructed a dual-targeting delivery system by using FA-modified M-β-CD to include adamantane-grafted HA (HA-AD). The resulting HA-Ad/FA-M-β-CD supramolecular complex could readily form nanosized assemblies with particle sizes (150.4 nm) favored for EPR effect. HA-Ad/FA-M-β-CD could be internalized into FR-α and CD 44-positive HCT 116 cells with significantly higher intracellular distribution compared to FA-M-β-CD. In addition, HA-Ad/FA-M-β-CD potently suppress the proliferation of FR-α and CD 44-positive HCT 116 cells. Unlike FA-M-β-CD-induced cell death via apoptosis, the dual-targeted HA-Ad/FA-M-β-CD complex induced cell death via mitophagy through impairment of the mitochondrial function. Furthermore, HA-Ad/FA-M-β-CD at a dose of 10 mg/kg significantly inhibited the tumor growth with negligible side effects in mice bearing HCT 116 human colon cancer xenograft [167].

For anticancer drug delivery systems, it is critically important to reduce the toxic effect on normal tissues. Xiao et al. designed a “plug and play”(PnP) light-sensitive polyanionic delivery system with the ability to inhibit normal tissues uptaking anticancer drugs. PAA (polyacrylic acid) grafted with Azo (azobenzene) (PAA-Azo) on its backbone could be recognized by α-CD through host–guest interaction. The trans form of Azo can be included in α-CD, while the cis form cannot be recognized. Because photoirradiation can trigger the transformation of the trans and cis forms of Azo [168], the release of α-CD modified drugs could be controlled using irradiation of UV light. When α-CD-modified drug such as an anticancer drug is loaded, the negatively charged polyanionic PAA-Azo will be repelled by the negatively charged normal cell surface. By using the irradiation of UV light at tumor tissues, anticancer drugs will be released and uptaken by tumor cells. This PnP-based delivery system can control the release of drugs at tumor sites to evade the toxic effects on normal tissues [169].

Thompson’s group developed a series of PVA-PEG (poly(vinyl alcohol)-poly(ethylene glycol)) pendant polymers consisting of pendant components such as adamantane or cholesterol to be included in β-CD via host/guest interactions. Cationic amino-β-CD or PEI-modified β-CD was used to incorporate the adamantane- or cholesterol-grafted PVA-PEG copolymer to form a supramolecular complex. The supramolecular complex enabled the compaction of gene agents such as pDNA or siRNA into stable nanoparticles that could be efficiently uptaken by target cells. In addition, these cationic CD/cholesterol or adamantane-modified PEG-PVA complexes were about 2~3 orders of magnitude less toxic than commercially available bPEI and capable of obtaining superior or comparable transfection efficiencies to those of bPEI or Lipofectamine 2000. Furthermore, acid-labile acetal linkage could be introduced into the pendant polymers to facilitate the degradation of the nucleic acid-compacted supramolecular complex at the acidic endosomes [170,171,172].

## 5. CD-Based Crosslinked Structures

### 5.1. Covalently Crosslinked Structures

Due to the high content of active hydroxy groups located in the exterior side, CDs are easily crosslinked to form reticulated structures. CDs can be crosslinked with polymers or directly cross-linked with crosslinker agents to produce network structures. The CD-based crosslink networks can further form hydrogel or nanosponges as anticancer drug carriers.

#### 5.1.1. CD Crosslinked with Polymers

Due to the abundant hydroxyl moiety that can be readily functionalized or crosslinked to form network structures, polysaccharides are widely used to covalently crosslink with CDs. Gami et al. crosslinked xylan (a class of hemicellulose) and β-CD to create a highly swellable hydrogel using EGDE (Ethylene Glycol Diglycidyl Ether) as a cross-linker. Curcumin and 5-FU (5-fluorouracil) could be entrapped into the hydrogel with drug loading of 26% and 98%, respectively [173]. As a naturally occurring polysaccharide approved by the FDA for wound healing and dietary applications, chitosan (CS) has been widely used for drug delivery. Modification of CS is a promising approach for the preparation of smart delivery systems. Wang et al. synthesized a 3D porous CS-grafted-β-CD (CS-g-β-CD) through Williamson ether synthesis reaction with epichlorohydrin (EPI) as the crosslinker. EPI was linked to β-CD via Williamson ether reaction, and the resulting EPI-β-CD was grafted to the primary amine of CS via nucleophilic reaction. Etoposide (VP16) was successfully entrapped in the CS-g-β-CD network with an encapsulation ratio of up to 73.6%. Interestingly, VP16 in the CS-g-β-CD exhibited a pH-sensitive profile. This might be due to the protonation of CS amino groups at acidic conditions, leading to the electrostatic repulsion between the positively charged CS chain, and further accelerating the release of encapsulated VP16. In addition, the release of VP16 markedly elevated with increasing temperature. The temperature-responsive property might be owing to the large amount of hydrogen bonds between hydroxyl groups and amino groups in CS-g-β-CD VP16. Heat absorption would be necessary to break the hydrogen bond when VP16 was released from the CS-g-β-CD carrier, thus leading to increased drug release with increasing temperature [174].

El-Zeiny et al. synthesized a β-CD-g-PNVCL (β-CD-grafted-N-vinylcaprolactam) copolymer by free radical grafting copolymerization with MBA (N, N’-methylene bisacrylamide) as a crosslinker. The β-CD-g-PNVCL nanogel was utilized as a carrier for 5-fluorouracil (5-Fu). The adsorption mechanism of 5-Fu by β-CD-g-PNVCL was excellently fitting with Langmuir model. In addition, 5-Fu loaded β-CD-g-PNVCL showed a controlled release behavior and the release continued for 30 h with 75% release in the gastric fluid and 68% release in the intestinal fluid. β-CD-g-PNVCL showed good biocompatibility with low toxicity to normal human lung fibroblast MRC-5 cells. In HCT-116 cells, 5-Fu-loaded β-CD-g-PNVCL exhibited enhanced inhibition effects in comparison with free 5-Fu [175].

Because of abundant amino groups existing in the polymer chain, PEI is capable of crosslinking with CDs to form a network structure. Ping and colleagues developed a redox-sensitive β-CD-crosslinked PEI to specifically target FGFRs (fibroblast growth factor receptor). CDI-activated β-CD was crosslinked with PEI; the resulting PEI-β-CD was linked to the FGFR-targeted MC11 peptide (MQLPLATGGGC) with SPDP (N-succinimidyl-3-(2-pyridyldithio) propionate) as a crosslinker to produce the MC11-PEI-β-CD (MPC) copolymer as host segment. PEG was conjugated with adamantyl group via a disulfide bond to generate the guest segment Ad-SS-PEG. The MPC/Ad-SS-PEG complex was able to condense pDNA efficiently and showed good stability against salt or BSA-induced aggregation. In FGFR-positive cell lines, gene transfection efficiency of MPC polyplexes was much higher than nontargeted polyplexes. The presence of disulfide bond could efficiently mediate endosomal escape and facilitate higher transfection efficiency. In addition, MPC/Ad-SS-PEG was capable of mediating tumor-targeted gene delivery in SKOV-3 tumor-bearing nude mice [176].

#### 5.1.2. CD as Monomer

**Figure 7:**
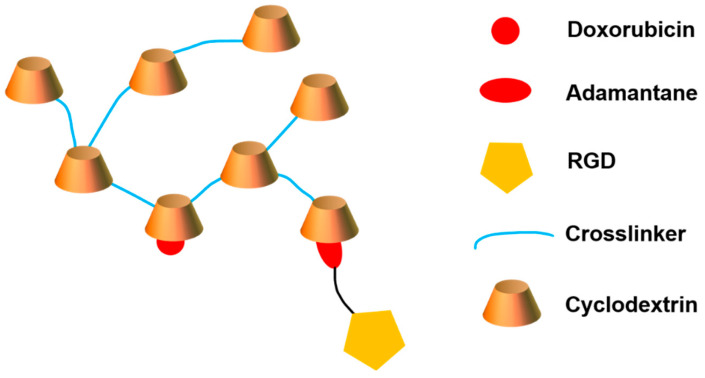
Schematic illustration of EPI-crosslinked γ-CD polymer complexed with AdRGD and Dox.

Epichlorohydrin (EPI) has been widely used as a crosslinker for CD crosslinking. Giglio and colleagues developed an EPI-crosslinked CD polymer to deliver sorafenib. The CD polymer significantly enhanced the water solubility of sorafenib, and the polymer-carried sorafenib showed a comparable antiproliferative effect to free sorafenib. In addition, the polymer-carried sorafenib markedly reduced the toxicity of free sorafenib in vivo [177]. Folic acid (FA) could be introduced to the crosslinked CD polymer to improve the tumor selectivity. Antitumor agent LA-12 (cis-trans-cis-[PtCl_2_(CH_3_CO_2_)_2_(adamantlyNH_2_)(NH_3_)]) was loaded into the CD cavity by host–guest interaction. The cytotoxicity of LA-12 was marked enhanced in the presence of FA-functionalized polymer compared to free LA-12 [178]. RGD (Arginine-glycine-Aspartate) is the smallest cell adhesion sequence that can be easily recognized by integrin receptors. Integrins are highly expressed in cancer cells. The activation of integrin will boost the downstream signaling pathways, facilitating cell proliferation and migration. RGD peptide-anchored carriers can be selectively recognized by integrin-overexpressed cells, accumulate at the target site and release the chemotherapeutics in a desired way to kill cancer cells [179]. Bognanni et al. developed an EPI-crosslinked γ-CD polymer functionalized with RGD peptide for integrin targeting (Figure 7). The crosslinked CD polymers could form stable nanoparticles with particle size of 20–30 nm. Dox was loaded into the CD polymers and the antiproliferative activity was significantly improved against HepG2 cell lines. In addition, the cytotoxic activity of Oxa (oxaliplatin) was also remarkably enhanced in the presence of CD polymers against HepG2 cells and A549 cells [180]. Viale and colleagues linked RGD peptide to an adamantane carboxylic moiety via a PEG_4_ chain to afford AdRGD. The AdRGD was included in the EPI-crosslinked CD polymers via host–guest interactions between CD and adamantane to introduce targeting units. The CD polymers could form nanosized assemblies, and Dox could be loaded into the assemblies. In comparison with free Dox, the RGD-decorated CD-crosslinked polymer-based carrier system markedly improved tumor selectivity and potentiated the cytotoxicity of the complexed Dox [181].

Citric acid is a polycarboxylic acid with good biocompatibility. Garcia-Fernandez et al. synthesized a β-CD polymer by crosslinking β-CD and citric acid. The obtained crosslinking polymer could be water-soluble or water-insoluble, with differences mainly in the preparation conditions and the degree of crosslinking [182]. Anand et al. developed two water-soluble citric acid-crosslinked γ-CD polymers (pγ-CD) with molecular weights of 21–33 kDa and 10–15 γ-CD units per molecule. The pγ-CD oligomers were capable of associating Dox with much higher binding constants compared to pβ-CyD. Additionally, the binding ability of pγ-CD for Dox was also larger than that of γ-CD with binding constants 1–2 orders of magnitude higher. The enhanced binding constant may be due to the inclusion of the bulky aglycone nucleus of Dox in the larger cavity and the electrostatic interactions between the positively charged Dox daunosamine segment and negatively charged citric acid crosslinker of pγ-CD. The pγ-CD-included Dox was efficiently uptaken into the nuclei of MCF-7 cells with DNA and nuclear protein as the binding sites [183].

Recently, a stimuli-responsive crosslink network was developed in response to microenvironmental stimuli to achieve controlled drug release. GSH-responsive delivery systems have become attractive since they enable the release of drugs predominantly inside cells. Several studies have reported the introduction of GSH responsiveness in CD-based nanosponges. These CD-based nanosponges were synthesized by reacting β-CD with 2-hydroxyethyl disulfide and the crosslinking agent pyromellitic dianhydride in a single step with a high yield up to 95%. Anticancer drugs such as Dox, strigolactone and resveratrol could be loaded into the GSH-responsive nanosponges with high loading content and encapsulation efficiency. The drug-loaded GSH-responsive nanosponges (GSH-NSs) were spherical in shape with average diameters of ~200 nm. In cancer cells with high GSH, the drug-loaded GSH-NSs displayed greater anticancer activities than free drugs (Dox, strigolactone analogues and resveratrol). In addition, GSH-NSs showed a good safety profile. Furthermore, the drug-loaded GSH-NSs was able to escape the P-gp efflux pump, which might be useful to overcome drug resistance for cancer treatment [184,185,186,187,188].

### 5.2. Crosslinked by Complex Interaction

Crosslinked network structures could be formed by host–guest interactions. Kovačević and colleagues reported a β-CD-grafted hyaluronan copolymer (CD-HA) modified via a triazole linkage with a substitution degree of 4%. Crosslinking adamantane-based guests could be entrapped in the hydrophobic CD cavity to form supramolecular networks. The CD units on CD-HA were able to form complexes with one, two or three-site adamantane-based guests. When multihead guests were incorporated into the CD cavity of the CD-HA chains, a supramolecular crosslinked network structure was formed [189]. Qian and coworkers developed a PAA-β-CD/PAA-TAX nanogel. β-CD and the hydrophobic anticancer drug PTX were grafted to the PAA main chain through ester or amide linkage; the resulting copolymer was subsequently crosslinked into nanogel via host–guest interactions. Drug leakage could be prevented due to the high stability of complex effects between β-CD and PTX, ensuring the controlled release of the drug to tumor sites. In addition, this PAA-β-CD/PAA-PTX nanogel showed strong adhesion to mucin due to the formation of hydrogen bonds with mucosa, leading to the prolonged drug retention in the vagina and further enhancing tumor inhibition in the aggressive U14 (cervical cancer cell) tumor model. Furthermore, the nanogel treatment significantly reduced side effects such as hepatotoxicity and nephrotoxicity that were obvious after PTX treatment [190].

Namgung et al. developed a nanoassembly that was formed via a host–guest interaction between polymer-CD conjugate (pCD) and polymer-PTX (pPTX) conjugate. Poly(IB-alt-MAnh) (poly(isobutylenealt-MAnh)) and poly(MVE-alt-MAnh) (poly(methyl vinyl ether-alt-MAnh) were used as the backbone polymer to conjugate CD and PTX through ester linkages. The multivalent inclusion complexes formed between pCD and pPTX readily formed nanoassemblies with an average radius of 54.6 ± 11.6 nm. The pPTX/pCD nanoassembly significantly enhanced the water solubility of PTX. In addition, the stability of pPTX/pCD was noticeably enhanced, with a 104-fold increase in Ka value in comparison with the PTX/CD monovalent complex. The high stability of the pPTX/pCD nanoassembly may prolong its circulation time and reduce the premature drug release, thus decreasing toxic effects to normal tissues. The cytotoxicity of pPTX/pCD nanoassembly was remarkably enhanced due to its high water solubility and accelerated PTX release by esterase after entering the cells. In MCF-7 cells, the IC50 of the pPTX/pCD nanoassembly was about 120-fold lower than that of free PTX. Furthermore, by functionalizing the nanoassembly with an AP-1 peptide that specifically target IL-4 (interleukin-4), the resulting pPTX/pCD nanoassembly could efficiently target aggressive and metastatic cells. The AP-1-grafted pPTX/pCD nanoassembly greatly enhanced tumor growth inhibition in the MDA-MB-231 xenograft mouse model compared to the nontargeted nanoassembly [191].

### 5.3. Crosslinking by Electrostatic Interaction

Ionic crosslinking is based on ionic interactions between polyanions and polycations. As a nontoxic crosslinking agent, citric acid-crosslinked CD polymers could serve as an anion source due to their abundant carboxylate groups. Karpkird and coworkers developed a nanoparticle system consisting of cationic chitosan and anionic citric acid-crosslinked β-CD. Cur was included in the cavity of crosslinked β-CD and further encapsulated into the nanoparticles (CSpβ-CD-cur). Cur-loaded nanoparticles were spherical in shape with mean particle sizes in the range of 298–314 nm. However, the cytotoxicity of CSpβ-CD-cur was less than free Cur, probably due to the sustained release of Cur from the nanoparticle [192].

## 6. Conclusions and Perspectives

Drug delivery systems with desired release profiles were developed to reduce the dosing frequency, decrease toxic effects on normal tissues and enhance efficacy to disease tissues. Controlled or sustained drug release can be achieved with CD-based polymeric carriers. The drug release rate and release site could be controlled by the type of polymers or CDs, the introduction of linkers, drug profiles and drug loading efficiency [193,194]. Drugs can be incorporated into nanoparticles formed by CD-based polymers or chemically conjugated onto CD copolymers with proper linkers. By introducing hydrophobic segments, CD-based polymers can easily form nanoparticles. Drugs loaded in nanoparticles usually showed sustained release profiles compared to free drugs. Yang and colleagues developed a CD-pullulan copolymer with hydrophobic glycyrrhetinic acid grafting on the polymer chains. The amphiphilic copolymers readily form spherical and uniformly sized particles in aqueous solutions. Dox was loaded into the nanoparticles and was released significantly slower than free Dox. Pharmacokinetic studies showed that the t _1/2_ of Dox-loaded NPs was 5.84-fold that of Dox.HCl. Interestingly, drug release from nanoparticles with higher Dox loading content was more sustainable than that of nanoparticles with lower drug loading content [195]. This might be due to the crystallization of Dox in the core of nanoparticles at high concentration [196]. In addition, drugs loaded in polymers with CDs have more than one loading mechanism in comparison with normal carriers. Hydrophobic drugs could be incorporated either in the hydrophobic domain of carriers (usually in the core of nanoparticles) or included in the cavity of CDs as inclusion complex. Therefore, drug release from CD-based polymeric carriers is likely to be more sustainable than normal delivery systems; Dox and Conferone (Conf)-loaded βCD-g-PMA-co-PLGA micelles exhibited a sustained release profile for about 14 days [162]. Bovine serum albumin-loaded nanoparticles formed by poly(DL-lactide-co-glycolide)-amino-CD conjugates showed triphasic release for 28 days, and the release rate could be controlled by the CD type and the monomer ratio [197]. When drugs were conjugated to CD polymers, a prolonged release could be achieved. The elimination half-life of NLG 207 was about 13.2 times higher than that of CPT alone in rats. The release of CPT in mice bearing NCI-H1299 was sustainable for more than 2 weeks. A similar result was observed in human patients [59,60,61]. PR-CPT conjugate showed a prolonged release profile for up to >7 days [14]. PR-PTX significantly prolonged the drug release, with t_1/2_ calculated to be about 5.8 h compared to 18.8 min reported for Taxol [18]. Stimuli-responsive bonds could be introduced to facilitate the release of chemotherapeutics at tumor sites. Drugs could be loaded into the polymeric carriers with stimuli-responsive linkages or chemically conjugated on polymers with stimulus bonds. By introducing stimulus groups (reduction-sensitive disulfide linkage, pH-sensitive hydrazone linkage and Schiff base, etc.) into the polymers, the polymeric carriers can rapidly release anticancer drugs into tumor tissues while slowly release them into normal tissues, leading to enhanced therapeutic effects and reduced side effects. In addition, the drug’s nature may also affect the release profile. Drug solubility and diffusivity are two important influence factors on the release rate of the drug [198]. For instance, Dox was more hydrophobic in a physiological solution due to the protonation of the amine group, while Dox was soluble in acidic media. Therefore, the release of Dox in a delivery system under a mildly acidic environment was much faster than that under physiological conditions [93,111].

CDs are known to improve the shelf life and stability of labile drugs. The formation of an inclusion complex may enhance the stability of drug molecules preventing them from many degradation process. Antibiotics with β-lactams and tetracylines are unstable in aqueous solution. Benzylpenicillin undergoes a short plasma half-life. By forming inclusion complex with HP-β-CD, the degradation against oxidation, acid-catalyzed hydration and hydrolysis was greatly decreased [199,200]. Cur is unstable at basic solutions and is found to be degraded even at physiological conditions. Complexes with CDs greatly decrease the degradation of Cur, with only 20~40% degradation occurring at 8 h, while more than 70% of pure Cur degraded at the same time [201]. Similar to CDs, the CD cavities of polymeric CDs are free to include drug molecules, and the polymeric CDs can also increase the stability of active molecules. β-CD nanosponges could maintain the physiochemical stability of rosuvastatin for 3 months and showed better plasma stability compared to its suspension formulation and marketed tablets [202]. The hydrolysis of β-lactam of meropenem leads to a loss of biological activity. Popielec et al. developed a QaβCDp (quaternary amino βCD polymer) that can self-assemble nanoparticles with CMC (carboxymethylcellulose). Meropenem was encapsulated in the CMC-QaβCDp nanoparticles and the β-lactam hydrolysis was decreased for about 30% at room temperature and 63% at 4 °C [200,203]. An intact lactone ring of CPT is crucial for its anticancer activity. However, the lactone ring is highly susceptible to spontaneous hydrolysis to the inactive carboxylate form at a physiologic pH. NLG207 is a CD-PEG-CPT copolymer with CPT covalently conjugating on the CD-PEG linear copolymer backbone. By conjugating CPT at the 20-OH position with CD-PEG, the labile lactone ring of CPT is successfully stabilized in its active form. In addition, intact nanoparticles formed by NLG207 prevent CPT from enzymatic and chemical hydrolytic degradation. Consequently, only the active form of CPT is released in a controlled and sustained manner [54]. For other CD polymeric carriers that form nanoparticles, the hydrophobic core may also shield the premature hydrolysis of the lactone ring of CPT [74].

This paper reviews CD-based polymers that are used for anticancer drug delivery (summarized in Table 1). CDs are regarded as safe carriers for drugs or gene agents, and there are several approved CD-containing products in the market. However, drug carriers with multiple functions and target characteristics to improve the therapeutic efficacy and reduce the toxicity of anticancer drugs are in urgent need for clinical applications. In addition, poor pharmacokinetics, plasma membrane disruption and hemolytic effects greatly limit the application of CDs. By combining the advantages of CDs and polymers, smart functions and specific properties can be achieved and toxic problems might also be solved. Due to large amounts of hydroxy groups on the surface, CDs are susceptible to being functionalized with polymers. Additionally, the hydrophobic cavity of CDs easily includes a lipophilic segment that is conjugated on the polymer or part of the polymer through host–guest interaction. Furthermore, stimuli-responsive linkages (reduction-sensitive disulfide linkage, pH-sensitive hydrazone linkage and Schiff base, etc.) and tumor-targeting ligands such as folic acid and hyaluronic acid could be introduced to CD-based polymers through the functional groups on CDs or polymers. The construction of an intelligent delivery system can release anticancer drugs regularly and specifically correspond to stimuli in a tumor microenvironment, thus enhancing the therapeutic efficacy and decreasing side effects. However, the extremely complicated human body, individual differences and the constant changes in internal environment render the stimulation of the internal environment a huge challenge. Therefore, the targetability and intelligent release of drugs will be affected and the fate of the CD-based polymer delivery system in vivo is still uncertain. In addition, the chemical synthesis of CD-based polymers is usually on a laboratory scale with intricate synthetic processes, and using toxic reagents. The industrial scale-up and the production of a stable final product is still a great challenge. Despite the above-mentioned problems, we believe that combined efforts and interdisciplinary collaboration from biologists, chemists, material scientists, biotechnologists and engineers will resolve any challenges encountered during the development of CD-based polymeric anticancer delivery systems in future studies.

## Figures and Tables

**Table 1:** Comparison of CD-based polymeric drug delivery systems.

		Structure Characteristics	Drug Loading	Stimuli-Responsive	Targeting
Polyrotaxane		CD cavity occupied by polymers	Conjugated on polymer chains or CDs/encapsulated in the nanoparticles	Y ^b^	Y ^c^
CDP conjugate ^a^		Drug conjugated on CD polymer	Conjugated on CDs or polymers	Y ^b^	Y ^c^
Copolymer	Linear-shaped	Linear chains	Encapsulated in the micelles	Y ^b^	Y ^c^
	Star-shaped	CD as the core with multipolymer arms grafted on it	Encapsulated in the micelles or included in the CD cavity		
	Brush-shaped	CDs grafted on polymer chains or complex with guests grafted on polymer chains	Encapsulated in the nanoparticles or included in the CD cavity		
Crosslinked polymer	Covalent crosslinking	CD as monomer or crosslinked with polymers	Encapsulated in the network or included in the CD cavity	Y ^b^	Y ^c^
	Complex crosslinking	Crosslinked by complex effect	Conjugated on the polymer		
	Electrostatic crosslinking	Crosslinked by electrostatic effect	Included in the CD cavity		

^a^ CDP conjugate: CD–drug polymeric conjugate; ^b^ stimuli responsiveness was achieved by introduction of stimuli-responsive linkages in the CD polymers; ^c^ targeting delivery was achieved by grafting of targeting moiety to CDs or polymers.

## Data Availability

Not applicable.
